# Neuronal *Gtf2i* deletion alters mitochondrial and autophagic properties

**DOI:** 10.1038/s42003-023-05612-5

**Published:** 2023-12-14

**Authors:** Ariel Nir Sade, Gilad Levy, Sari Schokoroy Trangle, Galit Elad Sfadia, Ela Bar, Omer Ophir, Inbar Fischer, May Rokach, Andrea Atzmon, Hadar Parnas, Tali Rosenberg, Asaf Marco, Orna Elroy Stein, Boaz Barak

**Affiliations:** 1 The Sagol School of Neuroscience, Tel Aviv University, Tel Aviv, Israel; 2 The School of Psychological Sciences, Faculty of Social Sciences, Tel Aviv University, Tel Aviv, Israel; 3 The Shmunis School of Biomedicine & Cancer Research, Faculty of Life Sciences, Tel Aviv University, Tel Aviv, Israel; 4 Neuro-Epigenetics Laboratory, Faculty of Agriculture, Food and Environment, The Hebrew University of Jerusalem, Rehovot, Israel

**Keywords:** Cellular neuroscience, Mechanisms of disease, Mitochondria, Developmental disorders, Molecular neuroscience

## Abstract

*Gtf2i* encodes the general transcription factor II-I (TFII-I), with peak expression during pre-natal and early post-natal brain development stages. Because these stages are critical for proper brain development, we studied at the single-cell level the consequences of *Gtf2i*’s deletion from excitatory neurons, specifically on mitochondria. Here we show that *Gtf2i*’s deletion resulted in abnormal morphology, disrupted mRNA related to mitochondrial fission and fusion, and altered autophagy/mitophagy protein expression. These changes align with elevated reactive oxygen species levels, illuminating *Gtf2i*’s importance in neurons mitochondrial function. Similar mitochondrial issues were demonstrated by *Gtf2i* heterozygous model, mirroring the human condition in Williams syndrome (WS), and by hemizygous neuronal *Gtf2i* deletion model, indicating *Gtf2i*’s dosage-sensitive role in mitochondrial regulation. Clinically relevant, we observed altered transcript levels related to mitochondria, hypoxia, and autophagy in frontal cortex tissue from WS individuals. Our study reveals mitochondrial and autophagy-related deficits shedding light on WS and other *Gtf2i*-related disorders.

## Abstract (web summary)

A neurobiological study reveals *Gtf2i’s* pivotal roles in brain development and mediating neuronal mitochondrial function, validated by human brain samples derived from individuals with Williams syndrome.

## Introduction

Proper brain development is accomplished through a precise sequence of developmental processes^1^, mediated mainly by the temporal and spatial regulation of gene expression. This is the result of transcription and transcription regulators such as the transcription factor called TFII-I, which is encoded by *GTF2I*. TFII-I is a vertebrate-specific multifunctional transcription factor, highly conserved and ubiquitously expressed. It is activated in response to a variety of extracellular signals and translocate to the nucleus^2–5^. As such, TFII-I can regulate the expression of genes^4,6,7^ through interactions with tissue-specific transcription factors and complexes related to chromatin remodeling^2,5^, and is capable of both promoting and inhibiting transcription^7^.

TFII-I was shown to be involved in various functions, among them regulating embryonic development^8–10^, cell cycle^2,6,11,12^, actin cytoskeleton and axon guidance^11^, endoplasmic reticulum stress response^13^, and epigenetic modulation^14–16^. TFII-I was also suggested to affect the excitatory/inhibitory balance of the brain^17^, a disruption known to be responsible for brain development impairments^18^. A study on human induced pluripotent stem cells showed that *Gtf2i* was responsible for 10–20% of the transcriptional dysregulation in pathways related to pathologies related to Williams syndrome (WS) and the 7Dup autism spectrum disorder^19^, both involved with altered expression of *GTF2I*. We recently studied the role of *Gtf2i* in post-natal excitatory neurons by deleting it using viral gene-editing techniques. This resulted in hyper-sociability, anxiety, impaired cognition, and hyper-mobility in young mice, suggesting *Gtf2i’*s importance in regulating behavior during the post-natal stage^20^. Together, these studies show that *Gtf2i* expression is critical for proper transcriptional regulation starting from early developmental stages and is essential for proper development of neural circuits involved in brain development and behavior^9,18,21–23^.

Altered transcriptional regulation, such as a result of malfunctioning transcription factor, may lead to cellular dysfunction, including defective energy production which is essential for cell viability and functionality. Due to the high energy demand of neurons, they are highly dependent on mitochondrial function to sustain their homeostasis^24,25^, support neural plasticity, and enable proper synaptic transmission^26^. Lack of sufficient energy support in neurons may therefore lead to defected neuronal plasticity and development, resulting in long-term changes in neural circuits activity.

In addition to providing essential energy for neurons, mitochondria maintain calcium balance, involved in metabolism of amino acids, lipids, and steroids, and generate free radicals, all are critical for neurons functionality^26–28^. To maximize functionality, mitochondria evolved as a cellular network that undergo fusion, or fission and biogenesis, in response to the ever-changing cellular environment, in order to meet the cells’ needs. This highly dynamic process is mediated by gene expression regulation at the transcription and translation levels, relying on tight coordination between the nuclear and mitochondrial genomes^29^. While the importance of mitochondrial properties in relation to proper neuronal development was observed by numerous studies, direct genetic evidence related to the role of TFII-I in mitochondrial health was never provided and is therefore the main aim of the current study.

Here, we chose to focus mainly on the consequences of *Gtf2i* deletion in forebrain excitatory neurons. Of the genes absent in WS, *Gtf2i* is of special interest as it has been linked in both humans^30–34^ and mice^20,22,23,35–38^ to the hypersociability and mental retardation associated with this condition. Because these impairments involve functions that are controlled by the cortex, a sub region of the forebrain, and because in the cortex excitatory neurons are major players in controlling the neuronal functions of the cortex, we studied in vitro primary excitatory neurons and the outcomes of *Gtf2i* deletion in those cells. Our in vitro studies revealed deficits in mitochondrial network morphology, alterations in molecular properties associated with mitochondrial fission and fusion, as well as autophagy and mitophagy, and increased levels of reactive oxygen species (ROS) at a single cell type resolution resulting from *Gtf2i* deletion in excitatory neurons. These alterations are further supported by our in vivo findings, as studied along the mouse cortex development.

To more accurately replicate the heterozygous deletion seen in individuals with WS, we developed a mouse model featuring heterozygous *Gtf2i* deletion and conducted subsequent in vitro investigations. Moreover, in order to gain deeper insights into the role of *Gtf2i* and its relevance to mitochondrial function, we explored the outcomes of a hemizygous neuronal *Gtf2i* deletion. Our findings unveiled meaningful mitochondrial alterations in both mouse lines, implying the presence of mitochondrial dysfunction in individuals with WS. These discoveries underscore the dosage-sensitive nature of *Gtf2i* and emphasize its crucial role as a key regulator of mitochondrial dynamics and overall mitochondrial health. Of clinical relevance, we confirmed mitochondrial abnormalities in terms of function, content, hypoxic levels, and autophagy in human frontal cortex brain samples from individuals with WS as compared to typically-developed (TD) controls. Together, our findings reveal new cellular pathways affected by *Gtf2i* deletion, and suggest new pathophysiological mechanisms that may explain neuronal dysfunction in WS and other *GTF2I*-related disorders.

## Results

### Basal levels of *Gtf2i* and TFII-I are highest at early developmental stages

**Fig. 1:**
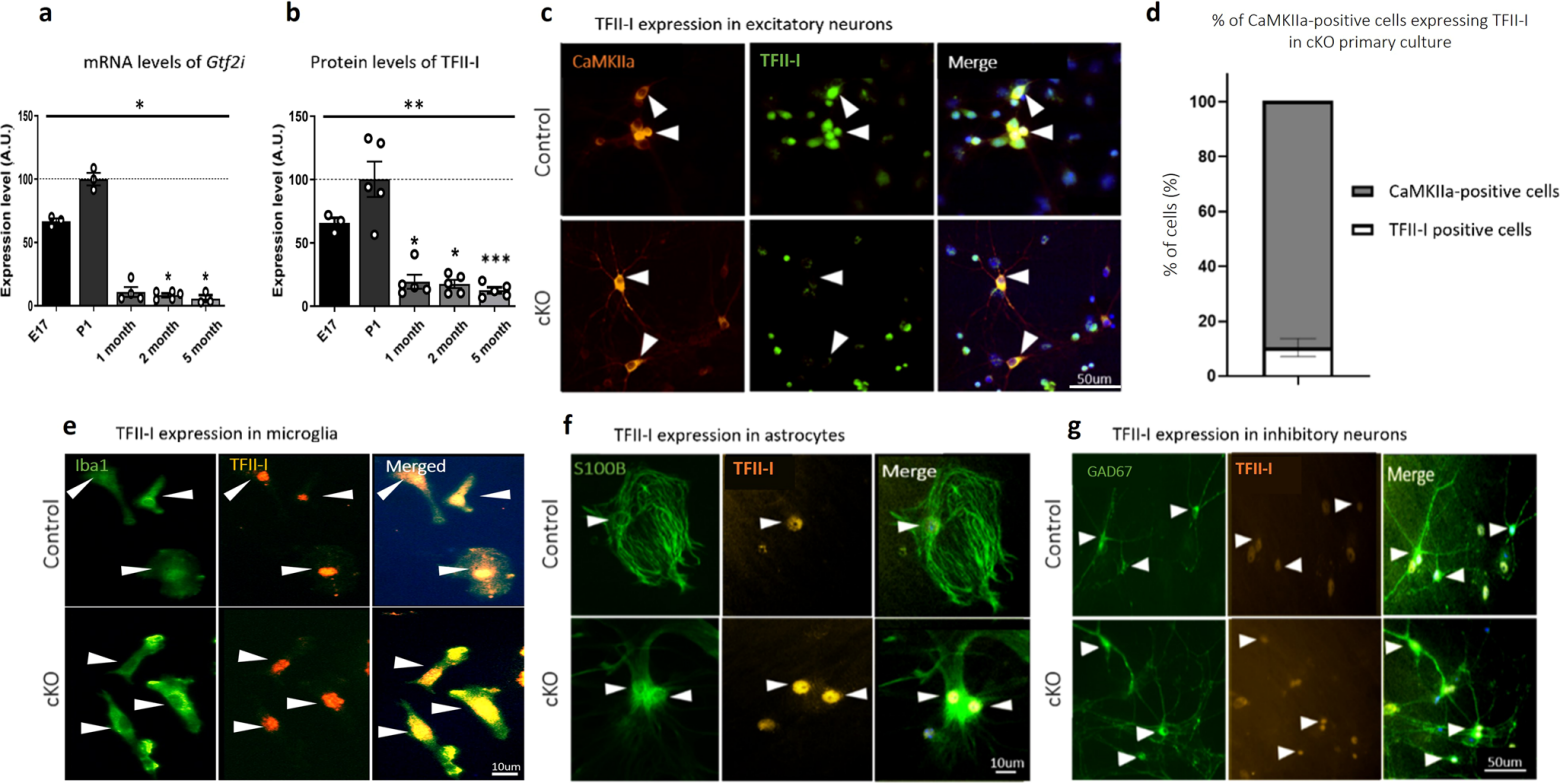
Basal levels of *Gtf2i* transcripts and TFII-I protein are highest at early developmental stages, while cKO mice demonstrate *Gtf2i* deletion specifically in excitatory neurons. **a** *Gtf2i* mRNA levels at E17 and at 1, 2, and 5 months of age, in comparison to post-natal day 1 (P1) (ns, ns, *P = 0.047, *P = 0.011, respectively). **b** TFII-I levels at E17 and at 1, 2 and 5 months of age, in comparison to P1, in whole hemispheres of naïve mice show the highest basal levels at P1 (ns, *P = 0.031, *P = 0.031, ***P = 0.003, respectively). **c** Representative images of primary cortical cultures from P1 cKO and control mice (days in vitro; DIV14) showing no TFII-I expression in excitatory neurons (identified by labeling with antibodies to CaMKIIa) of cKO mice. Arrowheads show excitatory neurons. **d** Quantitation of TFII-I expressing cells from all CaMKIIa-expressing cells shows that only 9.7% of excitatory neurons express TFII-I in cKO mice. Co-localization of **e** microglia (labeled with anti-Iba1 antibodies, see arrowheads), **f** astrocytes (labeled with anti-S100B antibodies, see arrowheads), and **g** inhibitory neurons (labeled with anti-GAD67 antibodies, see arrowheads) and TFII-I showed that TFII-I levels in glia cells and inhibitory neurons of cKO mice remain as in controls. Statistical significance was determined by **a**, **b** Kruskal–Wallis tests with multiple comparisons, **a** *n* = 3 for E17, *n* = 3 for P1, *n* = 4 for 1 month-, *n* = 5 for 2 month and *n* = 3 for 5 month-old mice; **b** *n* = 3 for E17, *n* = 5 for P1, *n* = 5 for 1 month-, *n* = 5 for 2 month-, and *n* = 5 for 5 month-old mice. **d** *n* = 3 for cKO mice, with 80–120 cells being studied from each mouse. Values represent means ± SEM. ns non-significant.

To explore *Gtf2i*-encoded TFII-I functions at its natural peak of expression, we first characterized *Gtf2i* mRNA transcript and TFII-I expression levels in whole hemispheres of naïve mice over the course of development. We noted the highest degrees of *Gtf2i* transcription and TFII-I expression in developing naïve mice to be in the pre-natal and early post-natal stages (Fig. 1a, b, respectively. Full data are described in the Supplementary Data 1), indicating important roles in mediating embryonic development^8,9,39^.

### *Gtf2i* deletion is specific to excitatory cortical neurons in cKO mice

These findings prompted us to study the role of *Gtf2i* at early post-natal stages, specifically focusing on neuronal functions. For this, we crossed *Gtf2i loxP* mice, which have *Gtf2i* floxed in both alleles^40^, with NEX-Cre mice^41^, a Cre line that selectively expresses Cre recombinase in excitatory neurons of the forebrain starting at embryonic day (E) 11.5. The resulting mice, subsequently referred to as cKO mice (*Gtf2i*^*f/f*^;NEX-Cre^+/−^), presented homozygous deletion of *Gtf2i* selectively in excitatory forebrain neurons^42^. These mice and their littermate controls (*Gtf2i*^*f/f*^;NEX-Cre^-/-^) were used to prepare primary cortical cultures, enabling us to study molecular and cellular properties at single cell resolution. As shown, TFII-I was absent in excitatory neurons of primary cKO cortical cultures (Fig. 1c), specifically from 90.3% of the excitatory neurons (Fig. 1d), yet was normally expressed in microglia (Fig. 1e), astrocytes (Fig. 1f), and inhibitory neurons (Fig. 1g).

### Neuronal *Gtf2i* deletion alters mitochondrial dynamics and morphology

**Fig. 2:**
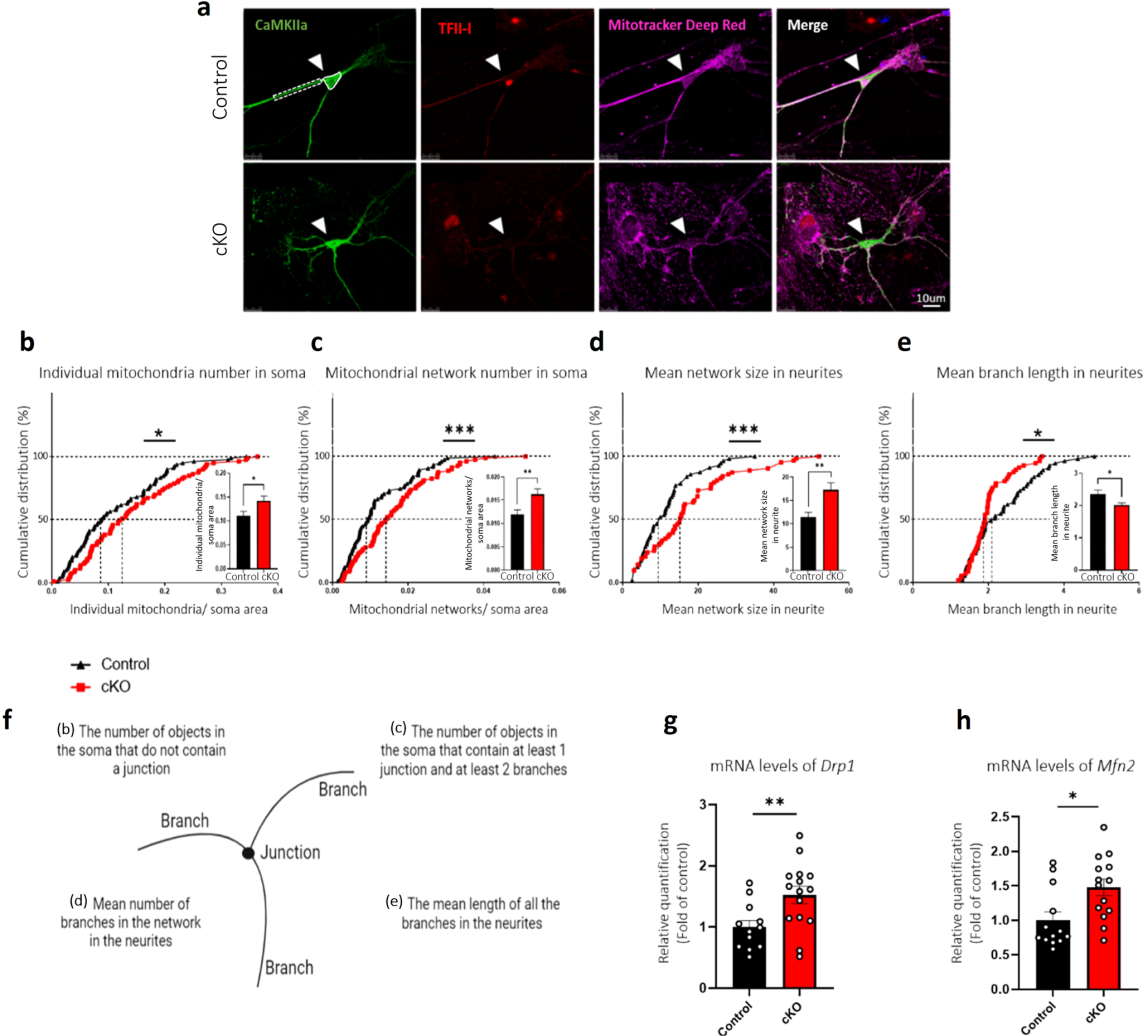
Mitochondrial network properties and morphology are altered in primary cortical cultures and whole cortex of cKO mice, as compared to controls. Primary cortical cultures from P1 cKO and control mice were seeded on coverslips. After 14 days, the cells were incubated with MitoTracker Deep Red followed by fixation and staining with anti-CaMKIIa and anti-TFII-I antibodies. **a** Representative images of neurons at DIV14. Arrowheads show excitatory neurons. Neuronal soma (surrounded by white continuous line) and neurites (surrounded by white dashed lines) were analyzed. Morphological skeleton analysis performed using the MiNA tool revealed **b** individual mitochondria numbers, normalized per soma area, to be higher in cKO mice (*P = 0.044), **c** increased numbers of networks, normalized per soma area, in cKO mice (***P = 0.003), **d** network sizes in neurites in cKO mice were significantly bigger (***P = 0.004) and **e** the mean length of mitochondria in networks in neurites were significantly shorter, reflecting smaller mitochondria, in cKO mice (*P = 0.048), all relative to controls. **f** A scheme of mitochondrial network parameters, as analyzed using MiNA. Parameters analyzed in panels b-e are listed in the scheme in parentheses. **g** Mitochondrial fission properties were measured by examining *Drp1 *mRNA transcript levels in homogenates of cortex from P1 cKO and control mice (**P = 0.009). **h** Mitochondrial fusion was measured by examining *Mfn2* mRNA levels in homogenates of cortex from P1 cKO and control mice (*P = 0.01). Statistical significance was determined by **b**–**e** Kolmogorov–Smirnov test or **g**, **h** Unpaired *t*-test. **b**–**e** *n* = 10 control mice, *n* = 10 cKO mice; 10–20 cells from each mouse. **g** *n* = 12 control mice, *n* = 15 cKO mice. **h** *n* = 13 control mice, *n* = 14 cKO mice. Values represent means ± SEM.

Mitochondria form interconnected networks to provide an efficient system for the delivery of energy, metabolites, and calcium channels to different areas of the cell^43,44^. These dynamic networks often change in shape and number, and are controlled by the fusion and fission of individual mitochondria. To study mitochondrial network dynamics as a result of *Gtf2i* deletion in excitatory neurons, we employed primary cortical cultures (Fig. 1c). Mitochondrial network morphology was visualized using MitoTracker Deep Red staining at DIV14 (Fig. 2a) and analyzed using the ImageJ MiNA macro tool^45^. Excitatory neurons were identified by staining with antibodies against CaMKIIa, and their soma and neurites were analyzed separately. MiNA was previously used to successfully detect and characterize morphological features of mitochondrial networks in cells in which mitochondria were labeled by MitoTracker staining^45^, including cells from primary cortical cultures^46,47^.

As shown in Fig. 2b, a lack of neuronal *Gtf2i* resulted in defective mitochondrial dynamics, as demonstrated by the significantly more individual mitochondria in the neuronal soma (normalized per area) in the deletion cultures than seen in the controls. Increased numbers of mitochondrial networks were also observed in cKO mice neuronal soma (Fig. 2c), while the mean number of branches per network was found to be significantly higher in cKO mouse neuronal neurites (Fig. 2d), as compared to controls. Finally, the mean branch length (*i.e.*, the length of those lines that represent mitochondrial structures according to MiNA) was significantly shorter in cKO mouse neuronal neurites as compared to controls (Fig. 2e), suggesting altered mitochondrial structure in the former.

The observed alterations in mitochondrial size, number, and shape in excitatory neurons lacking *Gtf2i* (Fig. 2a–f) could be the result of altered fission and fusion dynamics, as these processes regulate mitochondrial network homeostasis^48,49^. Key proteins involved in mitochondrial fusion include the outer membrane GTPases mitofusin 1 and 2 (Mfn1 and Mfn2) and the inner membrane GTPase optic atrophy 1 (OPA1) that coordinate double membrane fusion with the activity of additional scaffolding proteins^50,51^. The key protein involved in mitochondrial fission is dynamin-related protein 1 (Drp1), which is crucial for the maintenance of mitochondrial morphology and dynamics^50,51^. To study how neuronal *Gtf2i* deletion affected mitochondrial fission and fusion dynamics in the mouse brain, we measured transcript levels of these components in whole cortex of P1 cKO mice, as compared to controls, using quantitative PCR (qPCR). We found that cKO mice had significantly higher *Drp1* mRNA levels in the whole cortex of P1 mice as compared to controls (Fig. 2g), suggesting altered fission-related properties in the mutant mice. To study molecular properties associated with fusion, we quantified *Mfn2* mRNA levels in the whole cortex of P1 mice and found significantly higher *Mfn2* transcript levels in cKO mice as compared to controls (Fig. 2h). The changes in mitochondrial gene expression levels observed in cKO mice could potentially be attributed to aberrant transcriptional regulation resulting from the absence of TFII-I in neurons. TFII-I has been demonstrated to interact with various DNA binding motifs^52^, suggesting its potential direct or indirect role in regulating the expression of these mitochondrial genes (Supplementary Fig. 1).

**Fig. 3:**
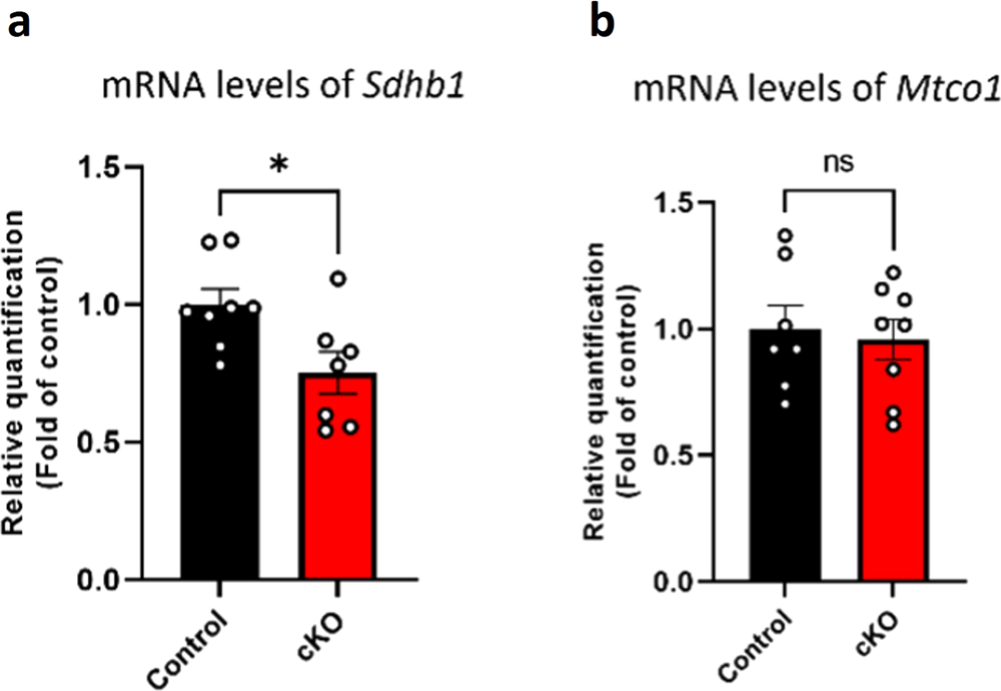
Mitochondrial functional properties are altered in P1 whole cortex of cKO mice, as compared to controls. **a** Mitochondrial functional properties were measured by examining *Sdhb1* transcript levels in homogenates of cortex from P1 cKO and control mice (*P = 0.02). **b** Mitochondrial content was measured by examining *Mtco1* levels (ns). Statistical significance was determined by unpaired *t*-test. **a** *n* = 8 control mice, *n* = 7 cKO mice. **b** *n* = 7 control mice, *n* = 8 cKO mice. Values represent means ± SEM. ns non-significant.

To further examine mitochondrial function and content as a result of neuronal *Gtf2i* deletion, we measured transcript levels of components associated with mitochondrial function and content in the whole cortex of P1 cKO mice, as compared to controls, using qPCR. We found that cKO mice had significantly lower mRNA levels of nuclear-encoded succinate dehydrogenase subunit B 1 (*Sdhb1*), a component of the mitochondrial respiratory chain (mitochondrial complex II), as compared to controls (Fig. 3a). *Sdhb1* is known for its function in the process of mitochondrial energy generation, thus, this downregulation may suggest altered mitochondrial function. Furthermore, we measured a marker of mitochondrial content, mitochondrially encoded cytochrome C oxidase I (*Mtco1*), in which no significant change was detected in the whole cortex of P1 cKO mice, as compared to controls (Fig. 3b).

### Neuronal *Gtf2i* deletion results in higher oxidative stress levels in primary cortical cultures compared to controls

**Fig. 4:**
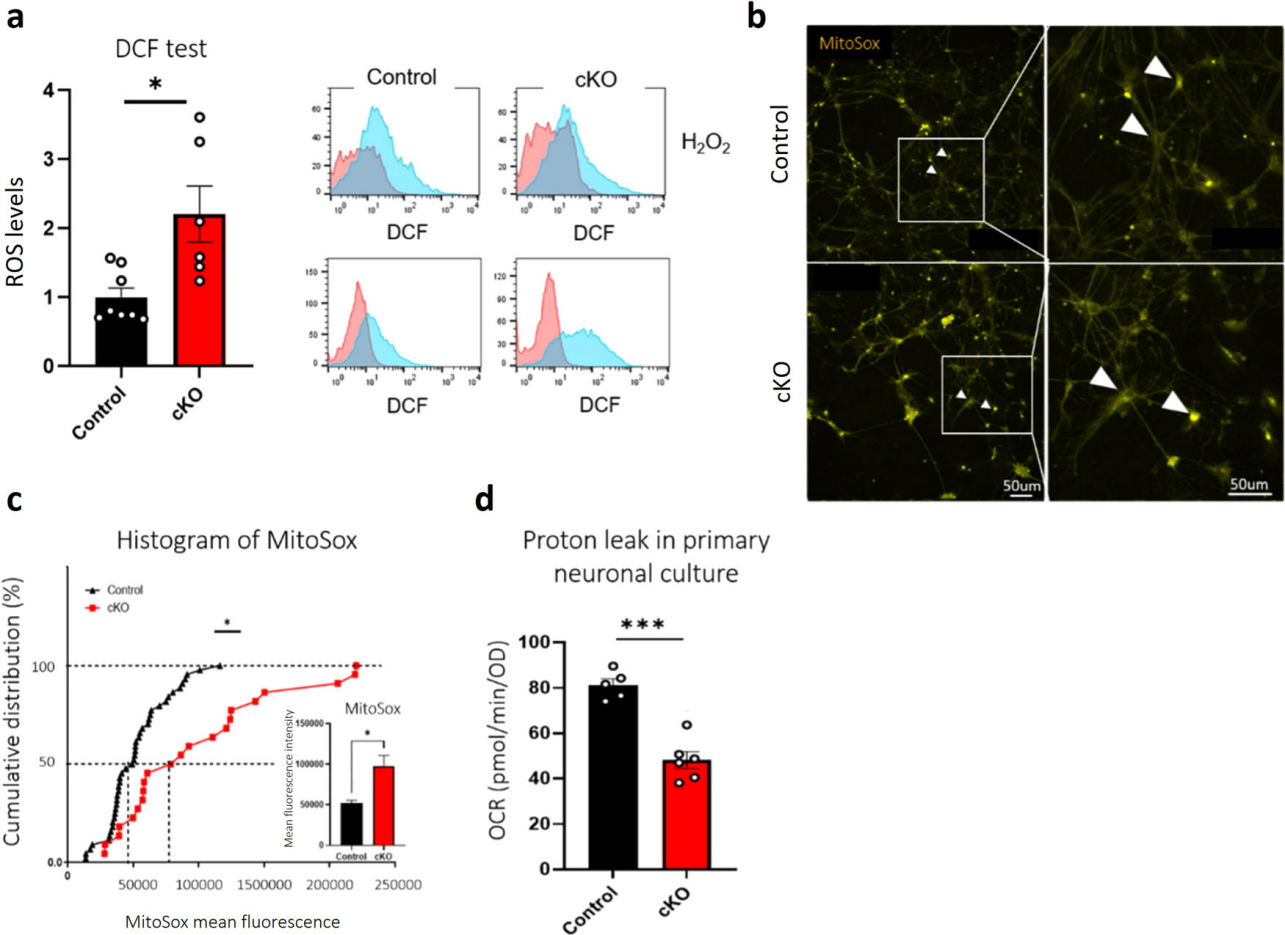
ROS levels are higher in cKO mice than in controls. Primary cortical cultures from P1 cKO and control mice were seeded and studied at DIV14. **a** ROS levels were examined by measuring DCF fluorescence by flow cytometry. H_2_O_2_ levels served as positive controls (*P = 0.01). **b** Mitochondrial ROS levels were measured using the MitoSox red reagent, specifically in the soma of excitatory neurons (arrowheads). **c** cKO mice showed higher ROS levels than did controls, as measured by assessing mean fluorescence intensity (*P = 0.014). **d** Primary cortical cultures from the cortex of cKO and control P1 mice were seeded on 96-well plates. Proton leak rates were determined at DIV14 using a Seahorse Mito-stress assay following pharmacological modifications and an XF96 analyzer. Results were normalized to cell number using a methylene blue assay according to the manufacturer’s instructions (***P < 0.0001). Statistical significance was determined by **a** Mann–Whitney test, **c** Kolmogorov–Smirnov test and **d** Unpaired *t*-test. **a** *n* = 8 control mice, *n* = 6 cKO mice, **c** *n* = 4 control mice, *n* = 3 cKO mice; 5–15 cells from each mouse. **d** *n* = 5 control mice, *n* = 6 cKO mice. Values represent means ± SEM.

A recent study suggested that aberrations in mitochondrial morphology, mitochondrial dysfunction, and high levels of ROS are interconnected^53^. Specifically, it was suggested that ROS plays a role in regulating mitochondrial morphology in the nervous system and specifically, in neurons^54^. To assess ROS levels in primary cultures prepared from the cortex of cKO mice, we performed a 2’,7’-dichlorodihydrofluorescein diacetate (DCF) fluorescent dye-based assay which relies on a ROS-sensitive probe to report on ROS levels in living cells. The DCF assay revealed ROS levels to be significantly higher as a result of neuronal *Gtf2i* deletion in the primary cKO cortical cultures, as compared to controls (Fig. 4a). To determine whether the higher ROS levels represent a by-product of abnormal mitochondrial respiratory function, we used the MitoSox red reagent, which is specifically targeted to mitochondria in living cells and emits fluorescence when oxidized (Fig. 4b). The finding confirmed that mitochondrial ROS levels were significantly higher in excitatory neurons derived from the primary cKO cortical cultures, as compared to controls (Fig. 4c).

To characterize molecular consequences associated with increased ROS levels in primary cortical cultures as a result of neuronal *Gtf2i* deletion, we next examined proton leak across the mitochondrial inner membrane. Proton leak can decrease ROS formation and, moreover, ROS can prompt proton leak, suggesting the existence of a feedback loop linking ROS and proton leak^55^. In addressing this putative relation, we found a significant reduction in proton leak in cKO mice, as compared to controls (Fig. 4d), suggesting that the negative regulation of ROS formation had been altered.

**Fig. 5:**
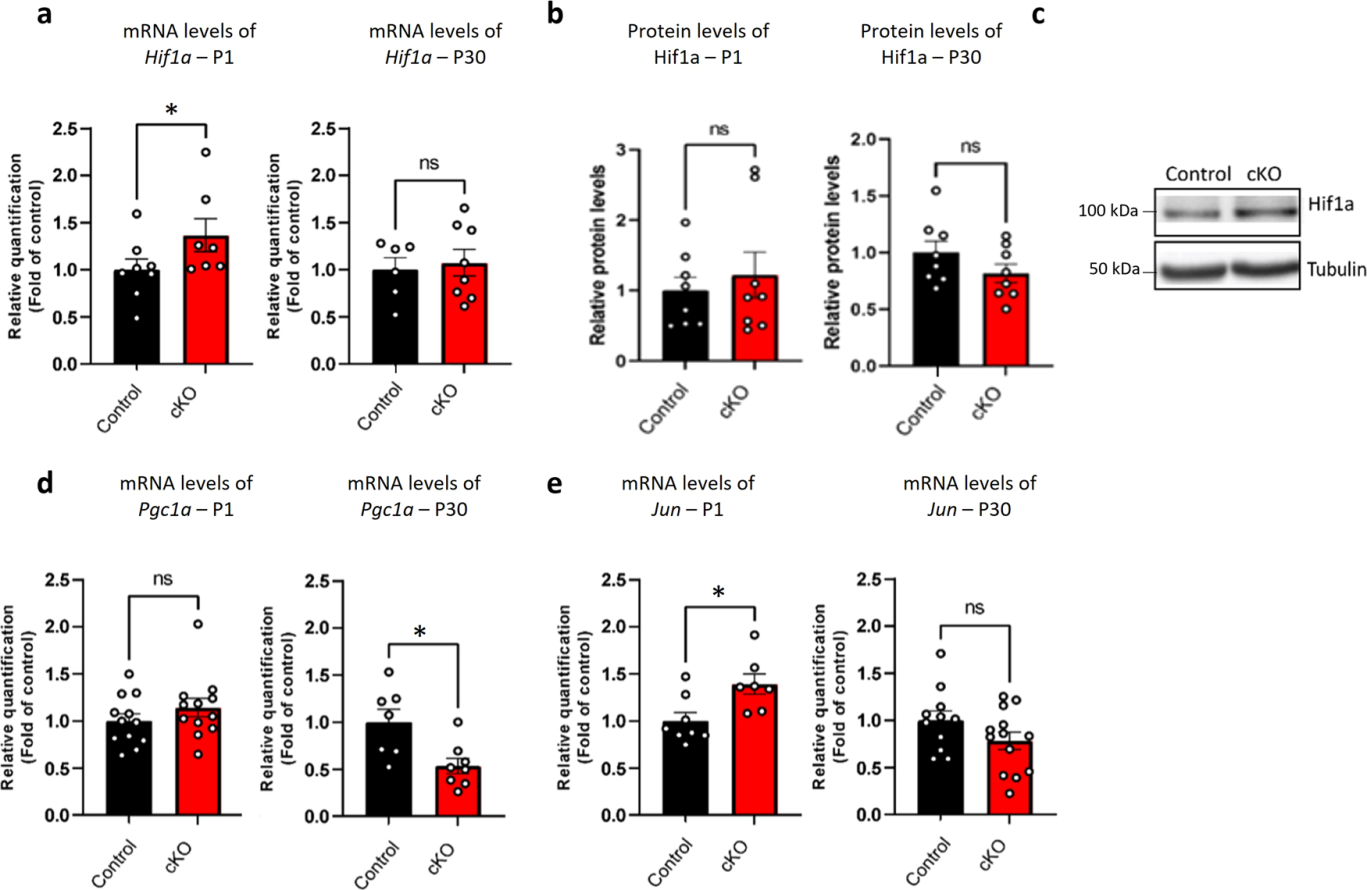
ROS and apoptotic-related markers are altered in the whole cortex of cKO mice compared to the controls. mRNA levels of ROS-related genes and protein levels in homogenates of cortex from cKO mice compared to control were examined. **a** Hypoxia-related properties were examined by measuring *Hif1a* mRNA transcript levels in homogenates of cortex from cKO and control mice. In P1 cKO mice showed significantly higher *Hif1a* mRNA levels (*P = 0.04), but not in P30 (ns), as compared to controls. **b** Whole cortex homogenates from P1 and P30 cKO compared to control samples were subjected to immunoblot, using the Hif1a antibody, and showed no significant difference in protein levels. **c** Representative results of the immunoblot of Hif1a. **d** ROS inhibition properties were examined by measuring *Pgc1a* mRNA levels in homogenates of cortex from cKO and control mice. In P1 cKO mice showed no significant difference (ns), while in P30 cKO mice showed significantly lower expression level of *Pgc1a* as compared to controls (*P = 0.01). **e** Apoptotic properties were examined by measuring *Jun* mRNA transcript levels in homogenates of cortex from cKO and control mice. In P1 cKO mice showed significantly higher *Jun* mRNA levels (*P = 0.01), but not in P30 (ns), as compared to controls. Statistical significance was determined by **a** Mann–Whitney test and **b**, **d**, **e** unpaired *t*-test. **a** *n* = 8 control mice, *n* = 7 cKO mice, *n* = 6 control mice, *n* = 8 cKO mice. **b** *n* = 8 control mice, *n* = 8 cKO mice. **d** *n* = 12 control mice, *n* = 12 cKO mice, *n* = 7 control mice, *n* = 8 cKO mice. **e** *n* = 8 control mice, *n* = 7 cKO mice, *n* = 11 control mice, *n* = 13 cKO mice. Values represent means ± SEM. ns non-significant.

We next measured the mRNA levels in the whole cortex in P1 and P30 mice, of ROS-related genes; hypoxia-inducible factor 1 alpha *(Hif1a)*, peroxisome proliferator-activated receptor gamma coactivator 1-alpha *(Pgc1a)* and apoptosis-related gene Jun proto-oncogene (*Jun*). *Hif1a*, which encodes a master regulator of cellular and systemic homeostatic response to hypoxia^56^, exhibited significantly higher mRNA levels at P1 in cKO mice (Fig. 5a), but no significant change in protein levels for Hif1a as compared to controls (Fig. 5b, c). There were no significant changes observed in mRNA and protein levels at P30 (Fig. 5a, b). Furthermore, *Pgc1a*, known for its role in inhibiting ROS production^57^ and acting as a master regulator of mitochondrial biogenesis^58,59^, exhibited significantly lower levels of mRNA expression in P30 cKO mice compared to controls, while no significant change was measured at P1 (Fig. 5d). *Jun*, known to be upregulated in response to oxidative stress^60,61^ and subsequently increase the release of the apoptotic Cyt-C from the mitochondria to the cytosol, exhibited significantly higher expression in cKO pups at P1 compared to controls. However, its expression normalized at P30 (Fig. 5e).

### Neuronal *Gtf2i* deletion results in decreased autophagy in primary cortical cultures under stress conditions

The altered mitochondrial properties, along with the increased ROS levels we observed due to neuronal *Gtf2i* deletion, prompted us to define a cellular mechanism that could explain these phenomena. One such mechanism is autophagy, namely, the process of defective organelles clearance^62^. In autophagy, LC3 (microtubule-associated protein 1 light chain 3) is converted into LC3-II (namely, the lipidated form of LC3), which then associates with the membrane of newly-generated autophagosomes, vesicles that engulf defective organelles, in this case defective mitochondria, destined for destruction^63^.

**Fig. 6:**
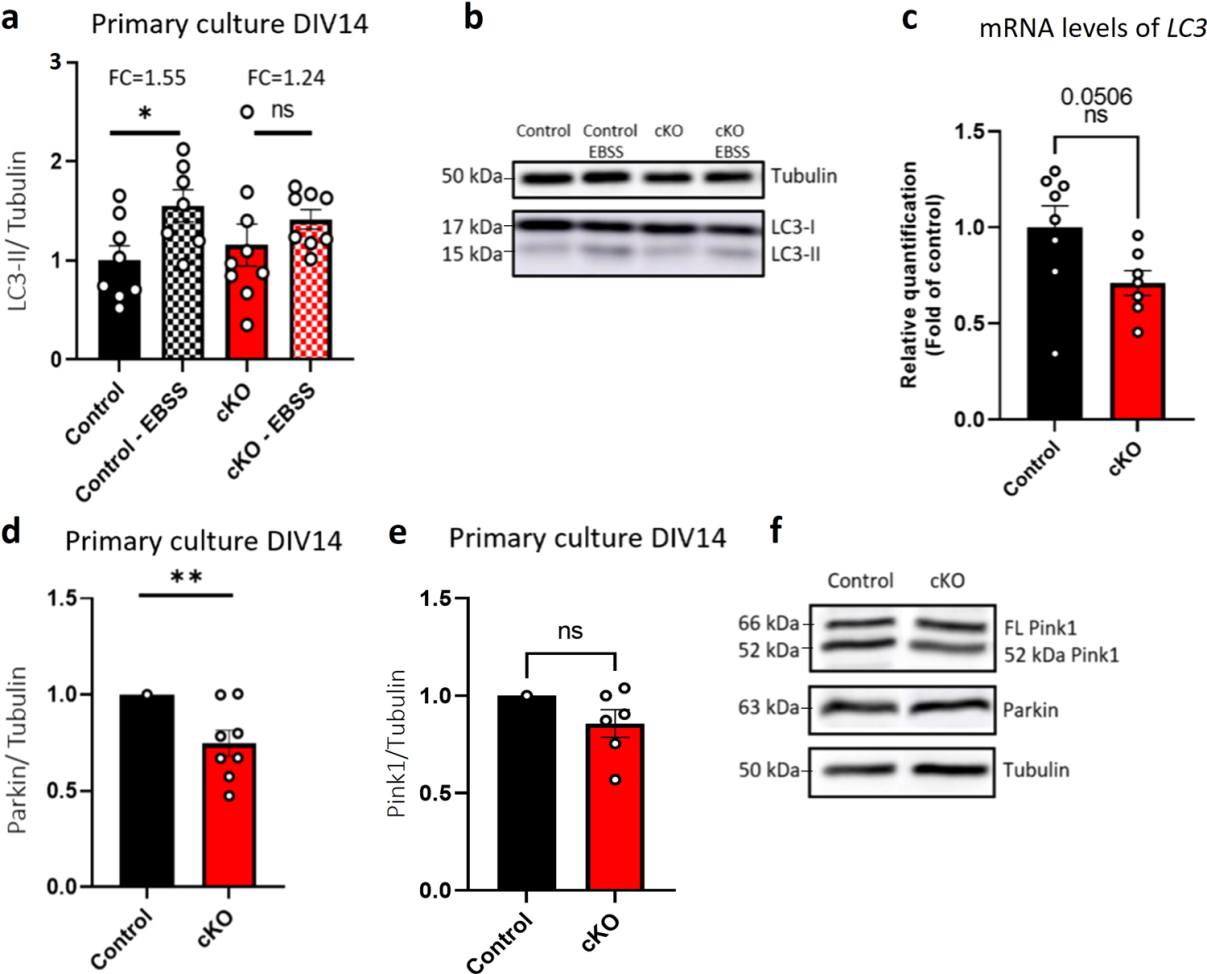
Autophagy and mitophagy levels are altered in cKO mice, as compared to controls. Primary cortical cultures from P1 cKO and control mice were seeded. After 14 days, proteins were separated by SDS-PAGE and subjected to immunoblot, using the indicated antibodies. **a** Autophagy was measured by examining the levels of LC3 protein in the primary cortical cultures. A significant increase in the LC3-II/Tubulin ratio was seen under stress condition (3 h EBSS starvation) in the case of control cells, yet no significant such changes were seen with cKO mouse cultures (*P = 0.025). **b** Representative results of anti-LC3 antibody staining. **c** mRNA levels of *LC3* are lower in homogenate cortex of cKO as compared to control mice at P1 (*P* = 0.0506). **d** Mitophagy was measured by examining Parkin protein levels. A significant reduction was detected in cKO cultures (**P = 0.006), while **e** assessing Pink1 protein levels revealed no significant changes (ns) between the two cultures. **f** Representative staining by the indicated antibodies. Statistical significance was determined by **a**, **c** Unpaired *t*-test and **d**, **e** One sample *t*-test. **a** *n* = 8 control mice, *n* = 7 control mice subjected to EBSS stress, *n* = 8 cKO mice, *n* = 8 cKO mice subjected to EBSS stress, **c** *n* = 8 control mice, *n* = 7 cKO mice. **d** *n* = 7 control mice, *n* = 8 cKO mice, **e** *n* = 7 control mice, *n* = 6 cKO mice. Values represent means ± SEM. ns non-significant, FC fold change, FL full length.

To characterize the extent of autophagy in primary cortical cultures, we induced Earle’s balanced salt solution (EBSS) starvation, a scenario that leads to autophagy^64^ and measured levels of the autophagic protein marker LC3. Under these stress conditions, we found significantly increased autophagy levels in control primary cultures (fold change (FC) = 1.55), as expected (Fig. 6a, b). However, in primary cKO cultures, there was no significant change in the response to stress (FC = 1.24), suggesting an inability to effectively induce autophagy (Fig. 6a) as a result of neuronal *Gtf2i* deletion.

To further characterize autophagy under neuronal *Gtf2i* deletion, we measured transcript levels of *LC3* in whole cortex of P1 cKO mice, as compared to controls, using qPCR. We found that cKO mice had lower *LC3* mRNA levels (*ns, P* = 0.0506) in the whole cortex of P1 mice as compared to controls (Fig. 6c), suggesting altered autophagy.

### Neuronal *Gtf2i* deletion alters mitophagy-associated protein levels in primary cortical cultures and transcript levels in whole cortex

The combination of mitochondrial dysfunction and decreased autophagy as a result of neuronal *Gtf2i* deletion prompted us to characterize mitophagy, a form of autophagy in which selective degradation of mitochondria occurs. One of the main pathways of mitophagy is the Pink1/Parkin pathway^65^. As such, we characterized Pink1 and Parkin protein levels in primary cultures and found a significant reduction in Parkin levels as a result of neuronal *Gtf2i* deletion, as compared to controls (Fig. 6d), which suggests a reduction in mitophagy in the cKO cultures. In contrast, Pink1 expression was unchanged (Fig.6e, f).

**Fig. 7:**
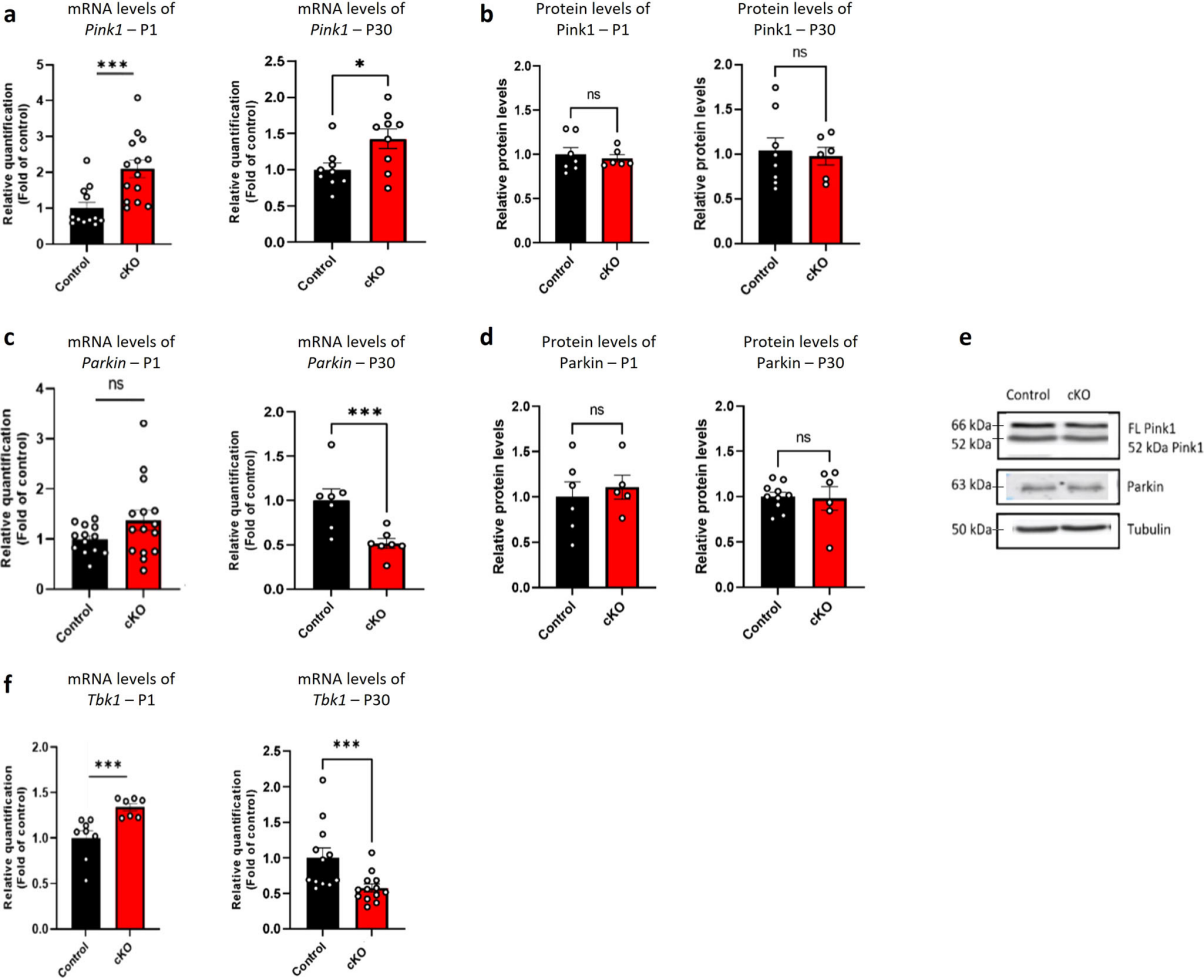
Mitophagy transcript levels are altered in the whole cortex of cKO mice compared to the controls. mRNA levels of mitophagy-related genes and protein levels in homogenates of cortex from cKO mice compared to controls were examined. **a** Mitophagy-related properties were examined by measuring *Pink1* mRNA transcript levels in homogenates of cortex from cKO and control mice. At P1 cKO mice showed significantly higher *Pink1* mRNA levels (***P = 0.0008) as well as at P30 (*P = 0.04), as compared to controls. **b** Whole cortex homogenates from P1 and P30 cKO compared to control mice were subjected to immunoblot, using the Pink1 antibody, and showed no significant difference in protein levels (ns). **c** *Parkin* mRNA levels in homogenates of cortex from cKO and control mice were measured. At P1 cKO mice showed no significant difference (ns), while in P30 cKO mice showed significantly lower expression level of *Parkin* compared to controls (***P = 0.004). **d** Whole cortex homogenates from P1 and P30 cKO compared to control samples were subjected to immunoblot, using the Parkin antibody, and showed no significant difference in protein levels (ns). **e** Representative results of the immunoblot of Pink1 and Parkin. **f** Mitophagy-related properties were examined by measuring *Tbk1* mRNA transcript levels in homogenates of cortex from cKO and control mice. P1 cKO mice showed significantly higher *Tbk1* mRNA levels (***P = 0.0003) and P30 showed significantly lower *Tbk1* mRNA levels (***P = 0.001), as compared to controls. Statistical significance was determined by Unpaired *t*-test/Mann–Whitney test. **a** *n* = 12 control mice, *n* =14 cKO mice, *n* = 9 control mice, *n* = 9 cKO mice, **b** *n* = 7 control mice, *n* = 6 cKO mice, *n* = 7 control mice, *n* = 6 cKO mice. **c** *n* = 13 control mice, *n* = 15 cKO mice, *n* = 7 control mice, *n* = 7cKO mice. **d** *n* = 6 control mice, *n* = 5 cKO mice, *n* =10 control mice, *n* = 6 cKO mice. **f** *n* = 8 control mice, *n* = 7 cKO mice, *n* = 12 control mice, *n* = 13 cKO mice. Values represent means ± SEM. ns non-significant. FL full length.

To further examine mitophagy and explore additional developmental stages, we conducted Western blot (WB) and qPCR experiments in whole cortex of P1 and P30 mice. We measured transcript levels of *Pink1* which revealed significantly increased amounts of *Pink1* mRNA levels in whole cortex of cKO mice at P1 and at P30, as compared to controls (Fig. 7a). We further measured Pink1 protein levels and did not find a significant difference between cKO mice and controls in P1 or P30 mice (Fig. 7b). We then measured transcript levels of *Parkin* and found significantly decreased levels of *Parkin* mRNA levels at P30, but no significant difference at P1 in cKO mice compared to controls (Fig. 7c). Parkin protein levels did not show a significant difference between cKO mice and controls (Fig. 7d, e). In addition, mRNA for *Tbk1*, which promotes mitophagy, was found significantly higher in cKO P1 mice, and significantly lower at P30 compared to controls (Fig. 7f).

#### Heterozygous and hemizygous mouse models result in altered mitochondrial morphology and higher oxidative stress levels in primary cortical cultures compared to controls

We observed various mitochondrial dysfunctions in a homozygous *Gtf2i* neuronal deletion model. To further validate the relevance of *Gtf2i* dosage-sensitive function, we developed a mouse model to replicate the heterozygous deletion of *Gtf2i* observed in individuals with WS.

**Fig. 8:**
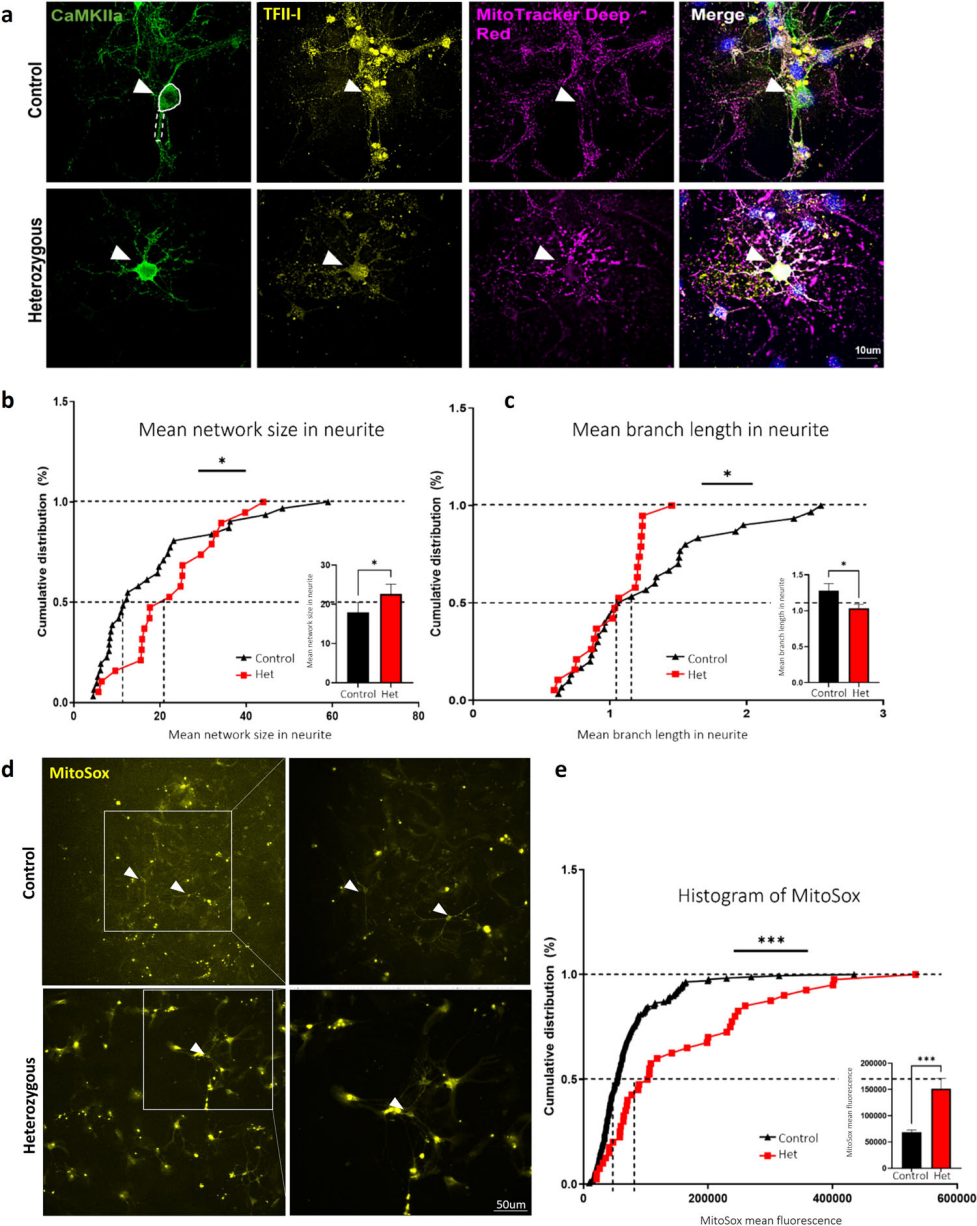
Mitochondrial network properties and ROS levels are altered in primary cortical cultures of *Gtf2i* heterozygous mice (*Gtf2i*^(+/-)^), as compared to controls. Primary cortical cultures from P1 *Gtf2i* heterozygous (*Gtf2i*^(+/-)^) and control mice were seeded on coverslips. After 14 days, the cells were incubated with MitoTracker Deep Red followed by fixation and staining with anti-CaMKIIa and anti-TFII-I antibodies. **a** Representative images of neurons at DIV14. Arrowheads show excitatory neurons. Neuronal soma (surrounded by white continuous line) and neurites (surrounded by white dashed lines) were analyzed. Morphological skeleton analysis performed using the MiNA tool revealed **b** network sizes in neurites in heterozygous mice were significantly bigger (*P = 0.02) and **c** the mean length of mitochondria in networks in neurites was significantly shorter, reflecting smaller mitochondria, in heterozygous mice (*P = 0.03), all relative to controls. **d** Mitochondrial ROS levels were measured using the MitoSox red reagent, specifically in the soma of excitatory neurons (arrowheads). **e** Heterozygous mice showed higher ROS levels than did controls, as measured by assessing mean fluorescence intensity (***P = 0.0003). Statistical significance was determined by Kolmogorov-Smirnov test. **b**, **c** *n* = 4 control mice, *n* = 4 heterozygous mice, 2–15 cells from each mouse. **e** *n* = 7 control mice, *n* = 5 heterozygous mice, 2-30 cells from each mouse. Values represent means ± SEM.

Mitochondrial network morphology was visualized using MitoTracker Deep Red staining at DIV14 (Fig. 8a) and analyzed using the ImageJ MiNA macro tool^45^. Excitatory neurons were identified by staining with antibodies against CaMKIIa, and their soma and neurites were analyzed separately. As shown in Fig. 8b, the heterozygous deletion of *Gtf2i* resulted in significantly higher number of branches per network in neuronal neurites as compared to controls. Additionally, the mean branch length was significantly shorter in heterozygous mouse neuronal neurites as compared to controls (Fig. 8c), suggesting altered mitochondrial structure.

To assess ROS levels in primary cultures prepared from cortex of heterozygous mice, we performed the MitoSox experiment (Fig. 8d). We found that mitochondrial ROS levels were significantly higher in excitatory neurons derived from the primary heterozygous cortical cultures, as compared to controls (Fig. 8e).

These findings provide compelling evidence for the importance of *Gtf2i* dosage in maintaining proper mitochondrial properties. Moreover, these results have important clinical implications for individuals with WS, as the heterozygous *Gtf2i* deletion closely resembles the genetic profile observed in individuals with WS^66^.

**Fig. 9:**
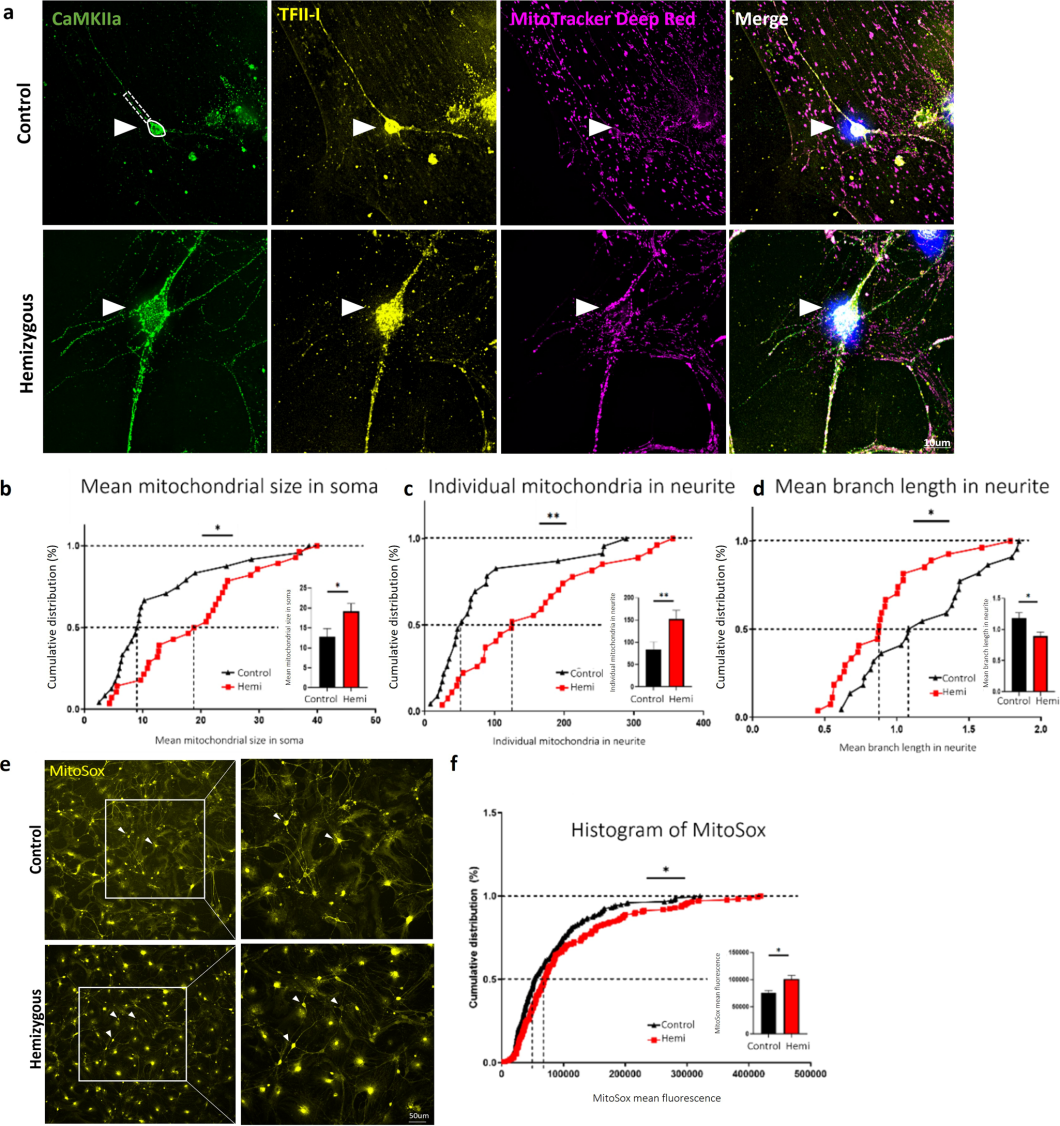
Mitochondrial network properties and ROS levels are altered in primary cortical cultures of hemizygous mice (*Gtf2i*^(*f*/+)^;*Nex*-Cre^(+/-)^), as compared to controls. Primary cortical cultures from P1 hemizygous (*Gtf2i*^(*f*/+)^;*Nex*-Cre^(+/-)^) and control mice were seeded on coverslips. After 14 days, the cells were incubated with MitoTracker Deep Red followed by fixation and staining with anti-CaMKIIa and anti-TFII-I antibodies. **a** Representative images of neurons at DIV14. Arrowheads show excitatory neurons. Neuronal soma (surrounded by white continuous line) and neurites (surrounded by white dashed line) were analyzed. Morphological skeleton analysis performed using the MiNA tool revealed **b** mean mitochondrial size in soma in hemizygous mice was significantly bigger (*P = 0.01), **c** individual mitochondria number in neurites was significantly higher (**P = 0.007) and **d** the mean length of mitochondria in neurites was significantly shorter, reflecting smaller mitochondria, in hemizygous mice (*P = 0.03), all relative to controls. **e** Mitochondrial ROS level was measured using the MitoSox red reagent, specifically in the soma of excitatory neurons (arrowheads). **f** Hemizygous mice showed significantly higher ROS level than did controls, as measured by assessing mean fluorescence intensity (*P = 0.02). Statistical significance was determined by Kolmogorov–Smirnov test. **b**–**d** *n* = 3 control mice, *n* = 3 hemizygous mice, 2–11 cells from each mouse. **f** *n* = 4 control mice, *n* = 4 hemizygous mice, 5–30 cells from each mouse. Values represent means ± SEM.

Next, we wanted to further explore whether the haploinsufficiency of *Gtf2i* specifically in forebrain excitatory neurons, is sufficient to induce mitochondrial dysfunction. To achieve this, we developed another mouse model of hemizygous *Gtf2i* neuronal deletion (*Gtf2i*^(*f/*+)^; Nex-Cre^(+/-)^). Mitochondrial network morphology was once again visualized using MitoTracker Deep Red staining at DIV14 (Fig. 9a) In this model we observed that the mean network size in the soma, as was measured by MitoTracker, was significantly increased (Fig. 9b) and that the number of individual mitochondria in the neurites was significantly higher, as compared to controls (Fig. 9c). We further found that the mean branch length of the mitochondria in the neurites was significantly shorter in hemizygous mouse neuronal neurites as compared to controls (Fig. 9d). This indicates a consistent effect of *Gtf2i* dosage on mitochondrial morphology, particularly in neurites.

Additionally, elevated ROS level was detected under the hemizygous neuronal deletion, as compared to controls (Fig. 9e, f), further emphasizing the similarities among all three mouse models we studied in this article, reinforcing the notion of *Gtf2i* as a key regulator of mitochondrial properties.

### Mitochondrial content, autophagy-related, and hypoxia-related transcripts expression levels in the brain are altered in individuals with WS

Our data suggest mitochondrial dysfunction as a result of neuronal *Gtf2i* deletion in mice, although mitochondrial properties in brain samples derived from individuals with WS as compared to TD controls are largely unknown.

To determine whether changes in mitochondrial properties occur in brain region relevant for the neurocognitive profile of WS, we extracted RNA from brain samples from Brodmann area 9 (BA9) sections, located in the frontal cortex, derived from individuals with typical deletion of the WS critical region (WSCR) and age-matched TD controls.

**Fig. 10:**
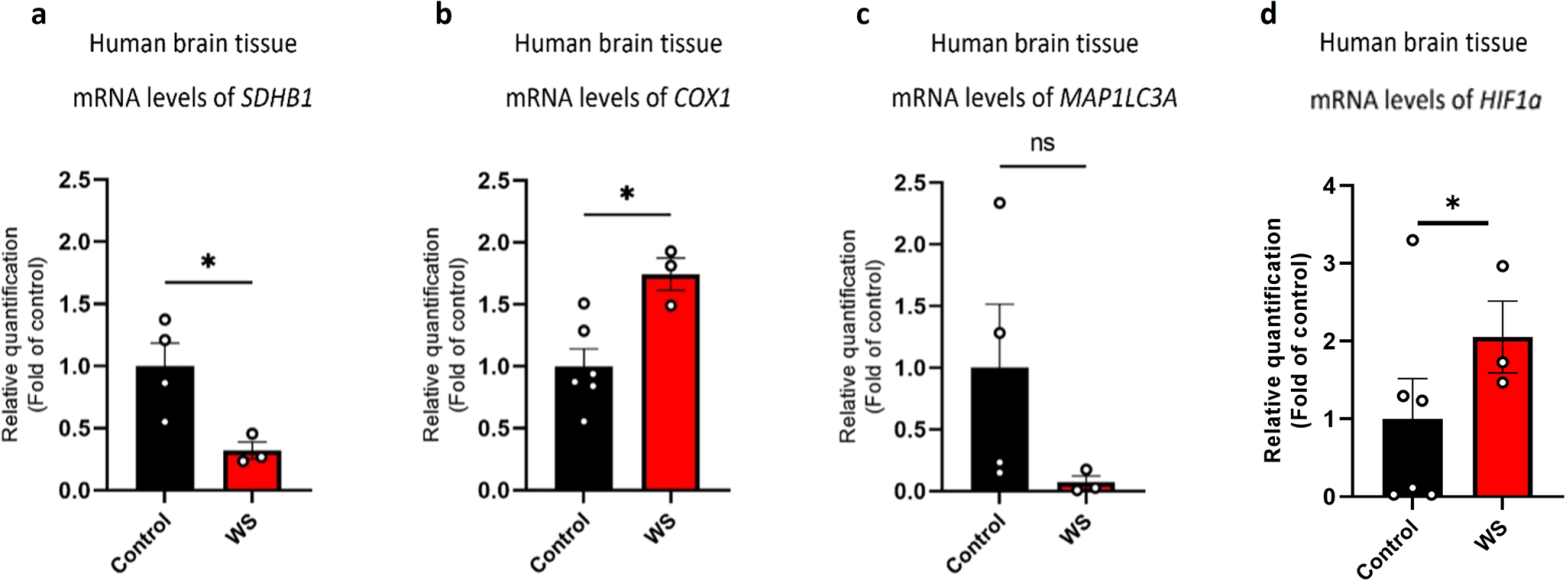
Abnormal mRNA expression levels associated with mitochondrial function, content, autophagy-related, and hypoxia-related transcripts in brain samples derived from individuals with WS, compared to TD controls. Human brain samples from frontal cortex BA9 sections obtained from individuals with typical deletion of the WSCR and age-matched TD controls were used, and their mRNA levels were quantified. Mitochondrial transcripts showed altered expression, with individuals with WS exhibiting **a** significantly lower levels of *SDHB1* mRNA (*P = 0.02), and **b** significantly higher levels of *COX1* mRNA (*P = 0.01) as compared to TD controls. **c** Autophagy-related transcript was measured by comparing the mRNA levels of *MAP1LC3A*, which showed lower levels in individuals with WS compared to TD controls (ns). **d** Hypoxia-related transcript was measured by comparing the mRNA levels of *HIF1a*, which showed significantly higher levels in individuals with WS compared to TD controls (*P = 0.01). Statistical significance was determined by Unpaired *t*-test. **a** *n* = 4 TD controls, *n* = 3 WS. **b** *n* = 6 TD controls, *n* = 3 WS. **c** *n* = 4 TD controls, *n* = 3 WS. **d** *n* = 6 TD controls, *n* = 3 WS. Values represent means ± SEM. ns non-significant.

Our transcriptomic analysis revealed significant decrease in *SDHB1* mRNA level in WS, as compared to TD controls, suggesting altered mitochondrial energy production (Fig. 10a). Additionally, individuals with WS showed a significant increase in *COX1* (the human gene name for the mouse gene *Mtco1*) mRNA level compared to TD controls, which is a marker for mitochondrial content (Fig. 10b). This confirms our findings of an increased number of mitochondria in our mouse model.

To study autophagy in the brain of individuals with WS, we measured the expression level of the autophagy-related *MAP1LC3A* transcript (known as *LC3* in mice) and found that the mRNA level was lower in WS as compared to TD controls, although not significantly (Fig. 10c), similar to the reduced expression in cKO mice compared to controls.

Last, to study if hypoxia-related properties are altered in the brain of individuals with WS, we measured the expression level of the hypoxic-related *HIF1a* transcript and found that the mRNA level was significantly higher in WS as compared to TD controls (Fig. 10d), which was also detected in the mouse model.

These transcriptional alterations may be explained by abnormal transcriptional regulation due to the haploinsufficiency of *GTF2I* (Supplementary Fig. 2). To further investigate the relationship between these genes, see Supplementary Fig. 3. Additionally, for an unbiased overview of transcriptional changes in relevant pathways see Supplementary Fig. 4.

## Discussion

Here, using primary cortical cultures and measuring mitochondrial properties, we sought to define *Gtf2i* roles in mitochondrial dynamics and function in excitatory neurons at the single-cell level. We provided several lines of evidence indicating that mitochondrial morphology and network are abnormal as a result of neuronal *Gtf2i* deletion, including an increased number of individual mitochondria and larger mitochondrial networks. These results suggest that TFII-I is critical in excitatory neurons for a balanced mitochondrial network and potentially in regulating energy distribution^67^. Moreover, we validated mitochondrial and autophagy-related deficits in human brain samples derived from individuals with WS, a neurodevelopmental disorder involved with *GTF2I* haploinsufficiency.

Defects in mitochondrial networks and morphology, such as we found, may be due to improper degradation of damaged mitochondria via mitophagy, which is crucial for energy homeostasis and mitochondrial function^67^. To investigate autophagy levels in our mouse model, we examined levels of LC3 protein in primary cortical cultures at basal levels and under stress conditions. We found that autophagy was not induced properly in neurons that lack *Gtf2i* expression under stress conditions as occurs in control cells. Stresses such as hypoxia, mitochondrial depolarization, or proteotoxicity can serve as a trigger for Pink1 accumulation on the mitochondrial surface and for recruitment of the ubiquitin E3 ligase Parkin, which in turn ubiquitinates outer mitochondrial membrane proteins to enable mitophagy^50,68–70^. Accordingly, the reduction in Parkin protein levels in cKO cultures, as compared to controls, suggests defective mitophagy, and may explain the increased number of individual mitochondria and larger mitochondrial networks we found in our model of neuronal *Gtf2i* deletion.

The defective mitochondrial morphology and decreased mitophagy that we measured in excitatory neurons unable to generate *Gtf2i* transcripts has the potential to affect oxidative phosphorylation in the defected neurons. ATP distribution over a wider cytoplasmic area could be facilitated by increasing mitochondrial length. Conversely, shorter mitochondrial lengths, as found for neurites in our model culture, could affect ATP distribution and lead to increased energy loss associated with respiration^71^. Moreover, shorter mitochondria could sequester ions less effectively over a given surface compared to longer mitochondria^67^.

The mitochondrial defects we described could also lead to generation of oxidative stress due to overproduction of ROS during the process of oxidative phosphorylation in the mitochondria^72^. Many different factors regulate ROS generation, including mild uncoupling of oxidative phosphorylation via proton leak through the inner mitochondrial membrane. Proton leak decreases ROS generation and ROS have been shown to induce proton leak, suggestive of a feedback loop between them^55^. In our mouse model, we showed reduced proton leak upon neuronal *Gtf2i* deletion, which may explain the up-regulation of ROS formation we detected, as a result of potential altered regulation.

To further characterize molecular aspects, we measured the transcript levels of *Hif1a*, a crucial regulator of cellular and systemic response to hypoxia, to assess whether hypoxia levels are higher due to neuronal *Gtf2i* deletion and elevated ROS levels. Interestingly, we observed elevated levels of *Hif1a* mRNA in the whole cortex of P1 cKO pups compared to controls, while protein levels remained unchanged. This finding suggests that following the complete deletion of neuronal *Gtf2i*, the increased levels of ROS may contribute to enhanced hypoxia.

Furthermore, we conducted an analysis of the transcript levels of *Pgc1a*, a recognized suppressor of ROS production, and identified a notable and statistically significant decrease. This prompted us to delve into the potential correlation between elevated oxidative stress and the upregulation of the apoptotic marker *Jun*. Subsequently, our findings substantiated this hypothesis. These discoveries enrich our comprehension of the intricate regulatory mechanisms governing cellular reactions to oxidative stress, encompassing phenomena like hypoxia and apoptosis. It is prudent to acknowledge that these genes harbor multifaceted roles beyond the scope of the observations outlined in this study.

The increased number of mitochondria we found as a result of neuronal *Gtf2i* deletion may lead to the increased ROS levels we measured, which, in turn, could lead to mitochondrial damage. It was previously shown that dysregulated ROS may contribute to processes that eventually lead to neurodegenerative diseases^72^ manifested by mtDNA mutations that cause neurological conditions^73^.

Our findings are supported by the recent suggestion that TFII-I*’*s targets are enriched in ROS-responsive genes^74^. Interestingly, previous studies suggested a mechanistic link between high ROS levels and the regulation of mitochondrial morphology and function, given that ROS regulates mitochondrial dynamics by affecting mitochondrial fusion and fission proteins^53,75^. More specifically, the balance between fusion and fission has been linked to ROS levels in neurons^76^.

To further validate the relevance of *Gtf2i* dosage, we sought to replicate the heterozygous microdeletion found in individuals with WS by inducing a heterozygous deletion of *Gtf2i*.

Our findings revealed substantial alterations in mitochondrial morphology and network, as well as an increase in ROS levels in these heterozygous mice, resembling the effects observed following the homozygous deletion of *Gtf2i* from excitatory neurons. These results provide compelling evidence for the involvement of *Gtf2i* in mitochondrial function and hold clinical relevance to individuals with WS, as the heterozygous *Gtf2i* deletion closely resembles the genetic profile observed in individuals with WS.

We proceeded with a mouse model in which *Gtf2i* was hemizygously deleted specifically from forebrain excitatory neurons to investigate whether the genetic haploinsufficiency of *Gtf2i* in these neurons alone is sufficient to induce mitochondrial dysfunction. Surprisingly, our findings once again demonstrated alterations in mitochondrial network and morphology, characterized by an increased number of individual mitochondria and mitochondrial networks. Furthermore, similar to the previous mouse lines studied in this research, we observed shorter mitochondrial branch lengths compared to the control group, and elevated levels of ROS in the excitatory neurons of hemizygous mice.

Notably, any type of *Gtf2i* deletion performed in this study led to significant changes, including increased mitochondrial number and shorter mitochondrial size. Moreover, the presence of elevated ROS levels, which are associated with altered mitochondrial function and can induce numerous neuronal alterations, was observed across all three mouse models. Mitochondrial dysfunction, and energy and metabolism deficits, are known features of numerous neurodevelopmental disorders^77,78^, including autism spectrum disorders^79–82^, Fragile X syndrome^83,84^, Angelman syndrome^84,85^, Rett syndrome^84,86^, and WS^87,88^. WS is caused by a heterozygous microdeletion of 25–27 genes^89,90^ on the long arm of chromosome 7 (7q11.23), which is part of the WSCR. WS individuals are known for their social abnormalities, particularly, they struggle with inhibiting their social behavior^91,92^, and exhibit non-social anxiety derived from fear and specific phobias^93^. WS individuals also have cognitive impairments^94,95^, with IQ scores typically ranging from 40 to 100, with a mean score of around 60^96^, which ranks them as mildly to moderately intellectually disabled.

To describe possible mitochondrial alterations in clinically relevant tissue samples, we examined mRNA levels in brain sections from the frontal cortex of individuals with WS and TD controls. Our data revealed reduced expression of *SDHB1* in individuals with WS as compared to TD controls. The encoded mitochondrially-localized protein is a subunit of the succinate dehydrogenase enzyme complex, responsible for transferring electrons from succinate to ubiquinone, critical for mitochondrial function as mediated by the mitochondrial respiratory chain complex II. The reduced expression of *SDHB1* in WS may decrease mitochondrial functionality in supporting cells energetically. Also, it may lead to increased ROS levels, similar to the increased ROS level we measured in our in vitro study, as a result of enhanced transfer of electrons to O_2_, due to the potential inhibited succinate-ubiquinone activity.

Studying additional mitochondria-related transcript, we found that *COX1* expression level in the frontal cortex of individuals with WS was higher, as compared to TD controls. *COX1* encodes the protein cytochrome c oxidase I, which is the main subunit of the cytochrome c oxidase complex, part of the respiratory complex IV. This complex is the third and final enzyme, taking part in the electron transport chain of mitochondrial oxidative phosphorylation. COX is acting in creating the electrochemical gradient essential for ATP synthesis as part of the oxidative phosphorylation^97,98^. Therefore, it is possible that the increased expression of *COX1* in WS represents the cells’ compensation machinery in order to increase their energy-generating capacity^99^. Interestingly, high level of COX1 expression was previously found in defective mitochondria, suggesting that mitochondrial genes can continue to function and be expressed in high levels, although their products are not functional properly^100^. *COX1* is also a known marker for mitochondrial content, and the observed significant increase in its levels in individuals with WS aligns with the data obtained from the mouse model, in which mitochondrial numbers were higher. To characterize autophagy-related properties in the human brain, we studied *MAP1LC3A* and found that its expression level in the frontal cortex of individuals with WS was lower, as compared to TD controls, although not significantly due to the high variability in the control group. Microtubule-associated protein 1 light chain 3 alpha is involved in autophagy, by taking active part in the formation of autophagosomes^101,102^. The reduced expression of *MAP1LC3A* in WS may therefore result in insufficient degradation of unhealthy cells in the brain of individuals with WS, or that unnecessary or dysfunctional components in those cells will not be removed properly. Finally, to examine hypoxia-related properties in WS, we measured *HIF1a* transcript levels and found its expression level in the frontal cortex of individuals with WS was significantly higher, as compared to TD controls, as was also found in our mouse model. This implies that human individuals with WS may be suffering from higher ROS levels which eventually lead to hypoxia. Since *GTF2I*, a member of the WSCR, is known to be a key player in the neurocognitive profile of WS, our results may explain how a lack of *GTF2I* expression affects neuronal-mediated functions such as cognition, social behavior, and other WS-associated pathologies. Other important factors affecting cognition and behavior, traits which require intact levels of energy and metabolism, and which are altered in WS, are myelination and signal conduction in the brain^42^. Significant energy consumption by myelinated axons is devoted to axonal repolarization, axonal transport, and the maintenance of glial cells^103^. The mitochondrial alterations we defined as a result of *Gtf2i* neuronal deletion may therefore affect cognition through neuronal-specific mechanisms, but also through neuron-glia interactions, affecting not only gray matter, but also white matter properties essential for proper brain functionality^104^.

*Gtf2i* roles in mediating mitochondrial properties were unknown before our study, to the best of our knowledge. Nevertheless, previous WS-related studies suggested a connection between WS and mitochondrial abnormalities, involving other genes from the WSCR. For instance, *Dnajc30* deletion in WS model mice resulted in dysfunctional mitochondria and functional differences in the oxidative phosphorylation pathway^87^. This study suggested that the protein encoded by *Dnajc30* acts as a regulator of mitochondrial respiration through interaction with ATP synthase^87^. In a second study, the *WSCR16* gene was demonstrated to be localized to mitochondria in HeLa cells^88^, while an earlier study suggested that WSCR16 could be co-precipitated with mitochondrial proteins and mitochondrial 16S rRNA^105^.

While widely studied in the context of the hypersociability and the neurocognitive aspects in WS^23,30–32,35,42,106^, *Gtf2i* mutations and altered expression are also related to autism spectrum disorders caused by duplication of 7q11.23^107–111^ and cancer^112–115^. TFII-I was also found to be involved in three autoimmune diseases: rheumatoid arthritis^116^, primary Sjogren’s syndrome^117^, and systemic lupus erythematosus^118,119^. Taken together, these studies demonstrate the wide diversity of functions TFII-I has in mediating proper cellular properties, and its wide clinical relevancy. In combination with our findings from the mouse model and the human brain samples, it is therefore conceivable to test in future studies whether targeting mitochondrial and autophagy-related deficits might be a beneficial therapeutic strategy for treating WS and other *GTF2I*-related pathologies.

## Methods

### Mice

*Gtf2i loxP* mice^40^ underwent more than 15 generations of back-crossed to pure C57Bl/6J mice (stock no. 000664; The Jackson Laboratory). All experimental protocols conformed to the guidelines of the Institutional Animal Care and Use Committee of Tel Aviv University, Tel Aviv, Israel. All efforts were made to minimize animal suffering and the number of animals used. Each cage contained 2–5 mice, regardless of genotype, within a controlled environment of 23 °C and a 12-h light–dark cycle (lights on at 7:00, lights off at 19:00) with free access to food and water. NEX-Cre mice are in a C57Bl/6 background^41^. NEX-Cre mice have exhibited normal behavior and development, with Cre activity commencing around E11.5, primarily limited to the excitatory neurons of the forebrain, particularly in the cortex and hippocampus^41^. *Gtf2i*-Het mice displayed germline deletion of a single *Gtf2i* allele. To induce hemizygous deletion of *Gtf2i* in excitatory neurons, *Gtf2i* homozygous *loxP* mice were bred with wild-type C57Bl/6J mice, resulting in the creation of mice with a single floxed *Gtf2i* allele and one wild-type allele [*Gtf2i*^(*f*/+)^mice]. Subsequently, these mice were crossed with NEX-Cre mice, leading to the generation of mice [*Gtf2i*^(*f*/+)^; NEX-Cre^(+/-)^] with targeted hemizygous *Gtf2i* deletion exclusive to excitatory neurons.

All test mice were males. Therefore, the limitations of this article arise from the absence of considering the sex of the mice in the experimental design, potentially overlooking gender-specific differences in the results.

### Primary cortical cultures

Pups were genotyped on P1 by PCR. Then, brains were removed and transferred to a culture dish containing ice-cold sterile PBS. Olfactory bulbs, basal ganglia, and cerebellum were removed, and the cortex was isolated, collected in digestion solution (HBSS-HEPES, CaCl_2_, EDTA, DNAse, l-cysteine, and papain (Sigma-Aldrich)), cut into smaller pieces with sterile scissors and transferred to a conical tube for 20 min, with shaking in room temperature (RT). After transfer to a conical tube containing neuronal plating medium (Neurobasal A (Rhenium), FBS, SM1 (Enco), glutamax (Rhenium), and Penicillin-Streptomycin), the tissue was mechanically digested by trituration. Cells were pelleted by centrifugation (1000 rpm for 4 min), resuspended in plating medium and seeded on matrigel (Lapidot)-coated coverslips in 6-, 12-, 96-well plates. Cultures were maintained at 37 °C in a humidified atmosphere of 95% air, 5% CO_2_. One half of the medium was replaced with fresh growth medium (without FBS) every 72 h. FUDR (Merck) was added at DIV4-8.

### Immunofluorescent staining

Primary cortical cultures were washed with PBS, followed by 10 min fixation in RT with 4% paraformaldehyde (PFA) and then washed 3 times (5 min for each wash) with PBS. Blocking was performed with Triton X-100 in PBS and 2% normal goat serum (NGS) for 1 h at RT. Primary antibodies diluted in blocking buffer were applied for 1 h at RT. Wells were then washed 3 times with PBS for 10 min each time and stained with secondary antibodies conjugated to Alexa Fluor (1:1000) in blocking buffer for 1 h at RT. Wells were washed three times in PBS for 10 min each time, followed by 4′,6-Diamidine-2′-phenylindole dihydrochloride (DAPI; 1:1000, Sigma) staining in PBS for 10 min, and an additional 20 min wash in PBS. Vectashield hardset mounting medium (Zotal) was used to mount the coverslips on glass slides. Images were captured using a Leica TCS SP5 Olympus confocal microscope, and an Olympus IX83 inverted microscope. Commercial antibodies used included anti-TFII-I (1:1000; CST-4562S, Cell signaling), anti-CaM Kinase IIa (1:400; 50049, Cell signaling), anti-Iba1 (1:500; 234 006, SYSY) anti-S100B (1:600; S-2532, Sigma-Aldrich) and anti-Gad67 (1:1000; MAB5406, Millipore) antibodies.

### Analysis of mitochondrial network morphology

Primary cortical cultures were incubated with 250 nM MitoTracker Deep Red FM (MTDR, Invitrogen) for 30 min at 37 °C. Wells were washed with PBS, followed by fixation with 100% ice-cold methanol at −20 °C for 20 min. Immunofluorescent staining was performed as described below. Mitochondrial network morphology of individual cells was visualized with a Leica TCS SP5 confocal microscope (×63 magnification) and analyzed using the ImageJ MiNA macro tool^45^. Analysis was performed in excitatory neurons alone and normalized to soma area. Neurites were all analyzed at the same distance from soma and at the same length.

### Proton leak assessment

The Seahorse XF Cell Mito Stress Test Kit (Agilent) was utilized. Cells (20,000) were plated on matrigel-coated Seahorse assay plates, and analyzed after 14 days in culture. The assay medium was formulated by enriching Seahorse XF medium with precise quantities of sodium pyruvate (1 mM), glutamine (2 mM), and glucose (10 mM). The medium was then heated to 37 °C and adjusted to a pH of 7.4. The Agilent Seahorse XF Cell Culture Microplate was primed for the assay by replacing the growth medium with the prepared assay medium. This step is followed by an incubation period to ensure the cells are in optimal condition before commencing the assay. Normalization was performed to compare similar experiments across different 96-well plates. The results were normalized to cell number using a methylene blue assay according to the manufacturer’s instructions.

### ROS measurement using DCF and MitoSox

#### DCF

For flow cytometry analysis of DCF staining, 250,000 cells were plated on matrigel-coated 6-well plates. After 14 days, the cells were washed and loaded with 10 μM of the dye (Sigma-Aldrich, D6883) for 30 min at 37 °C. After washing to remove excess dye, the cells were suspended in trypsin for 5 min at 37 °C and then in PBS and centrifuged. As a control, 1 µM of H_2_O_2_ was introduced to the cells for 2 h at 37 °C.

#### MitoSox

Primary cortical cultures at DIV14 in 12-well plates were washed and incubated with 2.5 µM MitoSOX Red (M36008, Rhenium) and Hoechst (33342) for 10 min at 37 °C. Coverslips with live cells were captured using an Olympus IX83 inverted microscope. Coverslips were fixed with 4% PFA and stained, as described.

### Lysate preparation and immunoblot

Fourteen day-old primary cortical cultures were starved for 3 h with EBSS (Sigma-Aldrich) and then dissected. Protein contents were separated using 4% Laemmli loading buffer, boiled, and resolved by SDS–polyacrylamide gel electrophoresis through 10% and 15% gels. The separated proteins were electrophoretically transferred to a nitrocellulose membrane in transfer buffer (25 mM Tris-HCl, pH 7.5, 190 mM glycine, and 10% methanol, absolute). Membranes were blocked for 45 min in TBST buffer (0.05 M Tris-HCl, pH 7.5, 0.15 M NaCl, and 0.1% Tween 20) with 6% skimmed milk, and blotted overnight with the indicated antibodies in TBST buffer, followed by incubation with secondary antibodies linked to horseradish peroxidase. Immunoreactive bands were detected with enhanced chemiluminescence reagent. Commercial antibodies used included rabbit anti-Hif1a (1:1000, PA1-16601, Rhenium), rabbit anti-LC3 (1:1000; 12741, Cell signaling), rabbit anti-Pink1 (1:1000; ab23707, Abcam) mouse anti-Parkin (1:100; 4211, Cell signaling) and mouse anti-beta-tubulin (1:5000; AB44928, Abcam) antibodies.

### RNA extraction

Upon ice thawing, extracted and frozen cortices underwent homogenization in 1 mL of cold TRIzol reagent (Thermo Fisher Scientific) utilizing a handheld electric homogenizer (Pro Scientific). Following a 5 min incubation at RT, 200 μL of chloroform was introduced to each sample, with subsequent manual tube shaking for 15 seconds. After another 3 min incubation at RT, the tubes were centrifuged for 20 min at 4 °C (13,800 rpm; Eppendorf Centrifuge 5430 R). Upon the separation of the mixture into three layers, the uppermost clear layer containing RNA was transferred to a fresh tube, to which 1:1 (v/v) isopropanol was added to induce RNA precipitation. After brief shaking and a 5 min RT incubation, the tubes were subjected to a 15 min centrifugation at 4 °C (13,800 rpm) to precipitate the RNA. The ensuing steps included removal of isopropanol, two washes with 1 mL of 80% ethanol (Sigma-Aldrich) in DEPC-treated water (Biological Industries), followed by a 5 min 4 °C centrifugation. After ethanol removal, the tubes were air-dried for 15–25 min, and subsequently, 20–35 μL of DEPC-treated water was added to each tube. Final RNA concentrations were determined using a Thermo Scientific NanoDrop One device (Thermo Fisher Scientific).

### mRNA expression

Total RNA extraction was used as the starting material for mRNA complementary deoxyribonucleic acid (cDNA) synthesis. The reverse transcription of mRNA was performed employing random primers and a High-Capacity cDNA Reverse Transcription Kit (Thermo Fisher Scientific). The C1000 Touch thermal cycler (Bio-Rad) was employed with the following parameters: 10 min at 25 °C, 120 min at 37 °C, 5 min at 85 °C, followed by a final step at 4 °C until completion.

### Real-time mRNA quantification

mRNA levels were determined using a Fast SYBR Green PCR Master Mix (Thermo Fisher Scientific), according to the manufacturer’s instructions, and the Bio-Rad CFX Connect Real-Time PCR Detection System (Bio-Rad). Thermal cycler conditions were as follows: 20 seconds at 95 °C, 40 amplification cycles (3 seconds at 95 °C to denature, and 30 seconds at 60 °C to anneal and extend), and a melt curve: 60 °C for 5 seconds, and an increase of 0.5 °C every 5 seconds (including a plate read) until reaching 95 °C. Expression values were calculated based on the comparative cycle threshold method^120^. mRNA levels were normalized to those of glyceraldehyde 3-phosphate dehydrogenase (*Gapdh*) mRNA. mRNA levels are shown as fold change, relative to levels in the control group. Specific primers for the detection of mRNA levels were ordered from Hy Laboratories (see supplementary table 1) and diluted to 10 mM in DEPC-treated water according to the manufacturer’s instructions.

### Human brain samples

All human tissue samples were kindly provided by the NIH NeuroBioBank at the University of Maryland, School of Medicine (NBB request #1068), in accordance with all legal provisions and relevant ethical considerations. Blocks of fixed control and WS human cortical brain samples from BA9 were examined.

### Human RNA isolation, cDNA preparation, and real-time polymerase chain reaction

RNA from BA9 cortex was isolated according to Kumar et al.^121^ with minor modifications, briefly: 650 mg of fixed tissue was lysed in 400 µl TES buffer (500 mM Tris pH 8, 100 mM EDTA, 100 mM NaCl, 1%SDS, 500 µg/mL proteinase K) and decrossed in 56 °C for 3 h. RNA was isolated from the lysates using TRI Reagent (Molecular Research Center) according to the manufacturer’s instructions. RNA was reverse-transcribed to single-stranded cDNA by Super Script II Reverse Transcriptase (Thermo Fisher Scientific) and random primers were mixed with 1 µM gene-specific primers using a mix of reverse primers from the quantified genes; qPCR was performed in a CFX Connect Real-Time PCR Detection System (Bio-Rad) with PerfeCTa SYBR Green FastMix (Quanta BioSciences). Dissociation curves were analyzed following each real-time qPCR to confirm the presence of only one product and the absence of primer dimer formation. The threshold cycle number (Ct) for each tested gene (X) was used to quantify the relative abundance of that gene using the formula 2^-(Ct geneX – Ct standard)^. Tubulin (*TUBA1B*) was used as the standard for mRNA expression. Primers used for the real-time qPCR and gene-specific amplification are listed in supplementary table 2.

### Multi correlation analysis

Correlation analysis between a set of genes driven from major biological pathways of mitochondrial functions, differential DNA methylation, and differentially expressed genes (DEGs) was performed by GeneOverlap R package. First, we used ToppGene Suite database in order to compile lists of genes specifically associated with the following biological pathways: Mitochondrial fusion, Pink1-PRKN mediated mitophagy, Mitochondrial biogenesis, Response to oxidative stress, Mitophagy, Mitochondrial fission, Negative regulation of oxidative stress-induced cell death, Response to mitochondrial depolarization and Apoptotic mitochondrial changes. As a negative control, we employed a set of genes driven from the biological pathway Structural constituent of cytoskeleton, which is not directly associated with mitochondrial function.

Next correlation analysis was performed between these gene lists and DEGs or genes that were annotated to differential DNA methylation. Transcriptional data were obtained from human brain samples of individuals with WS^42^, and were presented as either DEGs calculated based on a *p*-value < 0.01 or false discovery rate (FDR) < 0.05. Further transcriptional data (DEGs, FDR < 0.05) were collected from brain samples of mice with *Gtf2i* homozygous deletion specifically from excitatory neurons^42^. Last, DNA methylation data were taken from human brain samples from individuals with WS^122^. *P* adjusted value (presented in numbers) and odds ratio (color scale) from Fisher’s exact are presented in Supplementary Fig. 3.

### Statistics and reproducibility

Data are presented as means ± standard error of the mean (SEM), as calculated by GraphPad Prism 8.4.3. *p*-values were calculated using Student’s *t*-test (two-sided unpaired *t*-test or one sample *t*-test), with *p* < 0.05 considered as significant (*<0.05, **<0.01, ***<0.005). Normality of distributions and equality of variances were checked and addressed accordingly using the appropriate statistical analysis. Outliers were determined via the extreme studentized deviate (ESD) method.

### Reporting summary

Further information on research design is available in the Nature Portfolio Reporting Summary linked to this article.

### Supplementary information

Supplementary Information

Description of Additional Supplementary Files

Supplementary Data 1

Reporting Summary

## Data Availability

All data and source data are included in the manuscript and the supplementary information and will be accessible to anyone interested upon reasonable request. The numerical source data for all graphs in the manuscript can be found in Supplementary Data 1. All uncropped blots images generated and analyzed during the current study are available in the supplementary information. All materials are commercialized and will be available upon reasonable request.

## References

[1] Jiang X, Nardelli J (2016). Cellular and molecular introduction to brain development. Neurobiol. Dis..

[2] Roy AL (2012). Biochemistry and biology of the inducible multifunctional transcription factor TFII-I: 10 years later. Gene.

[3] Roy AL (2007). Signal-induced functions of the transcription factor TFII-I. Biochimica et Biophysica Acta (BBA)-. Gene Struct. Expr..

[4] Cheriyath V, Desgranges Zp Fau Roy AL, Roy AL (2002). c-Src-dependent transcriptional activation of TFII-I. J. Biol. Chem..

[5] Roy AL (2001). Biochemistry and biology of the inducible multifunctional transcription factor TFII-I. Gene.

[6] Desgranges ZP (2005). Inhibition of TFII-I-dependent cell cycle regulation by p53. Mol. Cell. Biol..

[7] Hakre S (2006). Opposing functions of TFII-I spliced isoforms in growth factor-induced gene expression. Mol. Cell.

[8] Makeyev AV, Bayarsaihan D (2009). New TFII-I family target genes involved in embryonic development. Biochem. Biophys. Res. Commun..

[9] Enkhmandakh B (2009). Essential functions of the Williams-Beuren syndrome-associated TFII-I genes in embryonic development. Proc. Natl Acad. Sci. USA.

[10] Enkhmandakh B, Bitchevaia N, Fau -, Ruddle F, Ruddle F, Fau -, Bayarsaihan D, Bayarsaihan D (2004). The early embryonic expression of TFII-I during mouse preimplantation development. Gene Expr. Patterns.

[11] Chimge NO, Makeyev FhR, Bayarsaihan D (2008). Identification of the TFII-I family target genes in the vertebrate genome. Proc. Natl Acad. Sci. USA.

[12] Ashworth T, Roy AL (2009). Phase specific functions of the transcription factor TFII-I during cell cycle. Cell Cycle.

[13] Hong M (2005). Transcriptional regulation of the Grp78 promoter by endoplasmic reticulum stress: role of TFII-I and its tyrosine phosphorylation. J. Biol. Chem..

[14] Bayarsaihan D (2013). What role does TFII-I have to play in epigenetic modulation during embryogenesis?. Epigenomics.

[15] Tussie-Luna MI, D B, Fau SE, Fh R, Roy AL (2002). Physical and functional interactions of histone deacetylase 3 with TFII-I family proteins and PIASxbeta. Proc. Natl Acad. Sci. USA.

[16] Strong E (2023). DNA methylation profiles in individuals with rare, atypical 7q11. 23 CNVs correlate with GTF2I and GTF2IRD1 copy number. NPJ Genom. Med..

[17] Wang Y (2010). Dlx5 and Dlx6 regulate the development of parvalbumin-expressing cortical interneurons. J. Neurosci..

[18] Barak B, Feng G (2016). Neurobiology of social behavior abnormalities in autism and Williams syndrome. Nat. Neurosci..

[19] Sanders SJ (2011). Multiple recurrent de novo CNVs, including duplications of the 7q11.23 Williams syndrome region, are strongly associated with autism. Neuron.

[20] Ophir O (2023). Deletion of Gtf2i via systemic administration of AAV-PHP. eB virus increases social behavior in a mouse model of a neurodevelopmental disorder. Biomedicines.

[21] Adamo A (2015). 7q11.23 dosage-dependent dysregulation in human pluripotent stem cells affects transcriptional programs in disease-relevant lineages. Nat. Genet..

[22] Lucena J (2010). Essential role of the N-terminal region of TFII-I in viability and behavior. BMC Med. Genet..

[23] Sakurai T (2011). Haploinsufficiency of Gtf2i, a gene deleted in Williams Syndrome, leads to increases in social interactions. Autism Res..

[24] Katsenelson M (2022). IGF-1 receptor regulates upward firing rate homeostasis via the mitochondrial calcium uniporter. Proc. Natl Acad. Sci. USA.

[25] Ruggiero A, Katsenelson M, Slutsky I (2021). Mitochondria: new players in homeostatic regulation of firing rate set points. Trends Neurosci..

[26] Mattson MP, Gleichmann M, Fau -, Cheng A, Cheng A (2008). Mitochondria in neuroplasticity and neurological disorders. Neuron.

[27] Gleichmann M, Mattson MP (2011). Neuronal calcium homeostasis and dysregulation. Antioxid. Redox Signal..

[28] MacAskill AF, Atkin Ta Fau Kittler JT, Kittler JT (2010). Mitochondrial trafficking and the provision of energy and calcium buffering at excitatory synapses. Eur. J. Neurosci..

[29] Nisoli E (2004). Mitochondrial biogenesis as a cellular signaling framework. Biochem. Pharmacol..

[30] Antonell A (2010). Partial 7q11.23 deletions further implicate GTF2I and GTF2IRD1 as the main genes responsible for the Williams-Beuren syndrome neurocognitive profile. J. Med. Genet..

[31] Dai L (2009). Is it Williams syndrome? GTF2IRD1 implicated in visual-spatial construction and GTF2I in sociability revealed by high resolution arrays. Am. J. Med. Genet..

[32] Morris CA (2003). GTF2I hemizygosity implicated in mental retardation in Williams syndrome: genotype-phenotype analysis of five families with deletions in the Williams syndrome region. Am. J. Med. Genet..

[33] Tassabehji M (2005). GTF2IRD1 in craniofacial development of humans and mice. Science.

[34] Danoff SK, Taylor HE, Blackshaw S, Desiderio S (2004). TFII-I, a candidate gene for Williams syndrome cognitive profile: parallels between regional expression in mouse brain and human phenotype. Neuroscience.

[35] Borralleras C, Sahun I, Perez-Jurado LA, Campuzano V (2015). Intracisternal Gtf2i gene therapy ameliorates deficits in cognition and synaptic plasticity of a mouse model of Williams-Beuren syndrome. Mol. Ther..

[36] Li HH (2009). Induced chromosome deletions cause hypersociability and other features of Williams-Beuren syndrome in mice. EMBO Mol. Med..

[37] Osborne LR (2010). Animal models of Williams syndrome. Am. J. Med. Genet. C, Semin. Med. Genet..

[38] Segura-Puimedon M (2014). Heterozygous deletion of the Williams-Beuren syndrome critical interval in mice recapitulates most features of the human disorder. Hum. Mol. Genet..

[39] Fijalkowska I, Sharma D, Bult CJ, Danoff SK (2010). Expression of the transcription factor, TFII-I, during post-implantation mouse embryonic development. BMC Res. Notes.

[40] Enkhmandakh B (2016). Generation of a mouse model for a conditional inactivation of Gtf2i allele. Genesis.

[41] Goebbels, S et al. Genetic targeting of principal neurons in neocortex and hippocampus of NEX-Cre mice. *Genesis* **44**, 611–621 (2006). 10.1002/dvg.20256 17146780

[42] Barak B (2019). Neuronal deletion of Gtf2i, associated with Williams syndrome, causes behavioral and myelin alterations rescuable by a remyelinating drug. Nat. Neurosci..

[43] De Giorgi F, Lartigue L, Ichas F (2000). Electrical coupling and plasticity of the mitochondrial network. Cell Calcium.

[44] Karbowski M, Youle RJ (2003). Dynamics of mitochondrial morphology in healthy cells and during apoptosis. Cell Death Differ..

[45] Valente AJ, Maddalena LA, Robb EL, Moradi F, Stuart JA (2017). A simple ImageJ macro tool for analyzing mitochondrial network morphology in mammalian cell culture. Acta Histochem..

[46] Wang L (2022). Enhancing S-nitrosoglutathione reductase decreases S-nitrosylation of Drp1 and reduces neuronal apoptosis in experimental subarachnoid hemorrhage both in vivo and in vitro. Brain Res. Bull..

[47] Cymerys J, Chodkowski M, Slonska A, Krzyzowska M, Banbura MW (2019). Disturbances of mitochondrial dynamics in cultured neurons infected with human herpesvirus type 1 and type 2. J. Neurovirol..

[48] Zamponi N (2018). Mitochondrial network complexity emerges from fission/fusion dynamics. Sci. Rep..

[49] van der Bliek, A. M., Shen, Q. & Kawajiri, S. Mechanisms of mitochondrial fission and fusion. *Cold Spring Harb. Perspect. Biol.* **5**, a011072 (2013). 10.1101/cshperspect.a011072 PMC3660830 23732471

[50] Liu YJ, McIntyre RL, Janssens GE, Houtkooper RH (2020). Mitochondrial fission and fusion: a dynamic role in aging and potential target for age-related disease. Mech. Ageing Dev..

[51] Palmer CS, Osellame LD, Stojanovski D, Ryan MT (2011). The regulation of mitochondrial morphology: intricate mechanisms and dynamic machinery. Cell. Signal..

[52] Makeyev AV (2012). Diversity and complexity in chromatin recognition by TFII-I transcription factors in pluripotent embryonic stem cells and embryonic tissues. PLoS ONE.

[53] Willems PH, Rossignol R, Dieteren CE, Murphy MP, Koopman WJ (2015). Redox homeostasis and mitochondrial dynamics. Cell Metab..

[54] Cid-Castro C, Hernandez-Espinosa DR, Moran J (2018). ROS as regulators of mitochondrial dynamics in neurons. Cell. Mol. Neurobiol..

[55] Brookes PS (2005). Mitochondrial H(+) leak and ROS generation: an odd couple. Free Radic. Biol. Med..

[56] Sharp FR, Bernaudin M (2004). HIF1 and oxygen sensing in the brain. Nat. Rev. Neurosci..

[57] Luo C, Widlund HR, Puigserver P (2016). PGC-1 coactivators: shepherding the mitochondrial biogenesis of tumors. Trends Cancer.

[58] Cheng A (2012). Involvement of PGC-1α in the formation and maintenance of neuronal dendritic spines. Nat. Commun..

[59] Lehman JJ (2000). Peroxisome proliferator–activated receptor γ coactivator-1 promotes cardiac mitochondrial biogenesis. J. Clin. Investig..

[60] Martindale JL, Holbrook NJ (2002). Cellular response to oxidative stress: signaling for suicide and survival. J. Cell. Physiol..

[61] Su B (2008). Oxidative stress signaling in Alzheimer’s disease. Curr. Alzheimer Res..

[62] Kelekar A (2005). Autophagy. Ann. New York Acad. Sci..

[63] Liu H (2017). From autophagy to mitophagy: the roles of P62 in neurodegenerative diseases. J. Bioenerg. And Biomembr..

[64] Munafo DB, Colombo MI (2001). A novel assay to study autophagy: regulation of autophagosome vacuole size by amino acid deprivation. J. Cell Sci..

[65] Pickles S, Vigie P, Youle RJ (2018). Mitophagy and quality control mechanisms in mitochondrial maintenance. Curr. Biol..

[66] Kozel, B. A. et al. Williams syndrome. *Nat. Rev. Dis. Primers.* **7**, 42 (2021). 10.1038/s41572-021-00276-z PMC9437774 34140529

[67] Chang DT, Reynolds IJ (2006). Mitochondrial trafficking and morphology in healthy and injured neurons. Prog. Neurobiol..

[68] Harper JW, Ordureau A, Heo JM (2018). Building and decoding ubiquitin chains for mitophagy. Nat. Rev. Mol. Cell Biol..

[69] Matsuda N (2010). PINK1 stabilized by mitochondrial depolarization recruits Parkin to damaged mitochondria and activates latent Parkin for mitophagy. J. Cell Biol..

[70] Ordureau A (2014). Quantitative proteomics reveal a feedforward mechanism for mitochondrial PARKIN translocation and ubiquitin chain synthesis. Mol. Cell.

[71] Skulachev VP (2001). Mitochondrial filaments and clusters as intracellular power-transmitting cables. Trends Biochem. Sci..

[72] Angelova PR, Abramov AY (2016). Functional role of mitochondrial reactive oxygen species in physiology. Free Radic. Biol. Med..

[73] Angelova PR, Abramov AY (2018). Role of mitochondrial ROS in the brain: from physiology to neurodegeneration. FEBS Lett..

[74] Kopp ND (2020). Functions of Gtf2i and Gtf2ird1 in the developing brain: transcription, DNA binding and long-term behavioral consequences. Hum. Mol. Genet..

[75] Jendrach M, Mai S, Pohl S, Voth M, Bereiter-Hahn J (2008). Short- and long-term alterations of mitochondrial morphology, dynamics and mtDNA after transient oxidative stress. Mitochondrion.

[76] Knott AB, Perkins G, Schwarzenbacher R, Bossy-Wetzel E (2008). Mitochondrial fragmentation in neurodegeneration. Nat. Rev. Neurosci..

[77] Uittenbogaard M, Chiaramello A (2014). Mitochondrial biogenesis: a therapeutic target for neurodevelopmental disorders and neurodegenerative diseases. Curr. Pharm. Des..

[78] A O, U M, Lf B, A GC (2021). Energy metabolism in childhood neurodevelopmental disorders. EBioMedicine.

[79] Wen, Y. & Yao, Y. *Autism Spectrum Disorders* (ed A. M. Grabrucker) (2021). 34495622

[80] Anitha A (2012). Brain region-specific altered expression and association of mitochondria-related genes in autism. Mol. Autism.

[81] Haas RH (2010). Autism and mitochondrial disease. Dev. Disabil. Res. Rev..

[82] Rossignol, D. A. & Frye, R. E. Mitochondrial dysfunction in autism spectrum disorders: a systematic review and meta-analysis. *Mol. Psychiatry* **17**, 290–314 (2012). 10.1038/mp.2010.136 PMC3285768 21263444

[83] Griffiths KK (2020). Inefficient thermogenic mitochondrial respiration due to futile proton leak in a mouse model of fragile X syndrome. FASEB J..

[84] Ortiz-Gonzalez XR (2021). Mitochondrial dysfunction: a common denominator in neurodevelopmental disorders. Dev. Neurosci..

[85] Santini E (2015). Mitochondrial superoxide contributes to hippocampal synaptic dysfunction and memory deficits in angelman syndrome model mice. J. Neurosci..

[86] Valenti D (2017). Stimulation of the brain serotonin receptor 7 rescues mitochondrial dysfunction in female mice from two models of Rett syndrome. Neuropharmacology.

[87] Tebbenkamp ATN (2018). The 7q11.23 Protein DNAJC30 Interacts with ATP Synthase and Links Mitochondria to Brain Development. Cell.

[88] Koyama M, Sasaki T, Sasaki N, Matsuura Y (2017). Crystal structure of human WBSCR16, an RCC1-like protein in mitochondria. Protein Sci..

[89] Korenberg JR (2000). VI. Genome structure and cognitive map of Williams syndrome. J. Cogn. Neurosci..

[90] Osborne LR (1999). Williams-Beuren syndrome: unraveling the mysteries of a microdeletion disorder. Mol. Genet. Metab..

[91] Pober BR (2010). Williams-Beuren syndrome. New Engl. J. Med..

[92] Laws G, Bishop D (2004). Pragmatic language impairment and social deficits in Williams syndrome: a comparison with Down’s syndrome and specific language impairment. Int. J. Lang. Commun. Disord..

[93] Dykens EM (2003). Anxiety, fears, and phobias in persons with Williams syndrome. Dev. Neuropsychol..

[94] Mervis CB (2000). The Williams syndrome cognitive profile. Brain Cogn..

[95] Mervis CB, Klein-Tasman BP (2000). Williams syndrome: cognition, personality, and adaptive behavior. Ment. Retard. Dev. Disabil. Res. Rev..

[96] Bellugi U, Lichtenberger L, Mills D, Galaburda A, Korenberg JR (1999). Bridging cognition, the brain and molecular genetics: evidence from Williams syndrome. Trends Neurosci..

[97] Schmidt TR (2005). Rapid electrostatic evolution at the binding site for cytochrome c on cytochrome c oxidase in anthropoid primates. Proc. Natl Acad. Sci. USA.

[98] Wikstrom, M. K. Proton pump coupled to cytochrome c oxidase in mitochondria. *Nature* **266**, 271–273 (1977). 10.1038/266271a0 15223

[99] Barrett T (2001). A murine dopamine neuron-specific cDNA library and microarray: increased COX1 expression during methamphetamine neurotoxicity. Neurobiol. Dis..

[100] Terman A (2003). Mitochondrial recycling and aging of cardiac myocytes: the role of autophagocytosis. Exp. Gerontol..

[101] Suzuki H (2014). Structural basis of the autophagy-related LC3/Atg13 LIR complex: recognition and interaction mechanism. Structure.

[102] Cherra SJ (2010). Regulation of the autophagy protein LC3 by phosphorylation. J. Cell Biol..

[103] Harris JJ, Attwell D (2012). The energetics of CNS white matter. J. Neurosci..

[104] Nir, A. & Barak, B. White matter alterations in Williams syndrome related to behavioral and motor impairments. *Glia* **69**, 5–19 (2021). 10.1002/glia.23868 32589817

[105] Arroyo JD (2016). A genome-wide CRISPR death screen identifies genes essential for oxidative phosphorylation. Cell Metab..

[106] Grad, M. et al. Altered White Matter and microRNA Expression in a Murine Model Related to Williams Syndrome Suggests That miR-34b/c Affects Brain Development via Ptpru and Dcx Modulation. *Cells.* **11**, 158 (2022). 10.3390/cells11010158 PMC8750756 35011720

[107] López-Tobón, A. et al. *GTF2I* dosage regulates neuronal differentiation and social behavior in 7q11.23 neurodevelopmental disorders. *Sci Adv.* **9**, eadh2726 (2023). 10.1126/sciadv.adh2726 PMC10686562 38019906

[108] Malenfant P (2012). Association of GTF2i in the Williams-Beuren syndrome critical region with autism spectrum disorders. J. Autism And Dev. Disord..

[109] Morris CA (2015). 7q11.23 Duplication syndrome: physical characteristics and natural history. Am. J. Med. Genet. Part A.

[110] Osborne LR, Mervis CB (2021). 7q11.23 deletion and duplication. Curr. Opin. Genet. Dev..

[111] Mervis CB (2015). Children with 7q11.23 duplication syndrome: psychological characteristics. Am. J. Med. Genet. Part A.

[112] Higuchi, R et al. Primary driver mutations in GTF2I specific to the development of thymomas. *Cancers.* **12**, 2032 (2020). 10.3390/cancers12082032 PMC7466068 32722121

[113] Feng Y (2017). GTF2I mutation frequently occurs in more indolent thymic epithelial tumors and predicts better prognosis. Lung Cancer.

[114] Nathany S, Tripathi R, Mehta A (2021). Gene of the month: GTF2I. J. Clin. Pathol..

[115] Kim DW, Cheriyath V, Roy AL, Cochran BH (1998). TFII-I enhances activation of the c-fos promoter through interactions with upstream elements. Mol. Cell. Biol..

[116] Kim K (2016). Association-heterogeneity mapping identifies an Asian-specific association of the GTF2I locus with rheumatoid arthritis. Sci. Rep..

[117] Li Y (2013). A genome-wide association study in Han Chinese identifies a susceptibility locus for primary Sjögren’s syndrome at 7q11.23. Nat. Genet..

[118] Sun C (2016). High-density genotyping of immune-related loci identifies new SLE risk variants in individuals with Asian ancestry. Nat. Genet..

[119] Meng Y (2019). Association of GTF2I gene polymorphisms with renal involvement of systemic lupus erythematosus in a Chinese population. Medicine.

[120] Schmittgen TD, Livak KJ (2008). Analyzing real-time PCR data by the comparative C(T) method. Nat. Protoc..

[121] Kumar K, Oli A, Hallikeri K, Shilpasree AS, Goni M (2022). An optimized protocol for total RNA isolation from archived formalin-fixed paraffin-embedded tissues to identify the long non-coding RNA in oral squamous cell carcinomas. MethodsX.

[122] Trangle SS (2023). In individuals with Williams syndrome, dysregulation of methylation in non-coding regions of neuronal and oligodendrocyte DNA is associated with pathology and cortical development. Mol. Psychiatry.

