# Supplementary material for: Neuronal Gtf2i deletion alters mitochondrial and autophagic properties
Source: Commun Biol. 2023 Dec 14;6:1269. doi: 10.1038/s42003-023-05612-5 (PMC10721858; doi:10.1038/s42003-023-05612-5)
Supplement: Supplementary file 1 — Supplementary Information [file 42003_2023_5612_MOESM1_ESM.pdf]

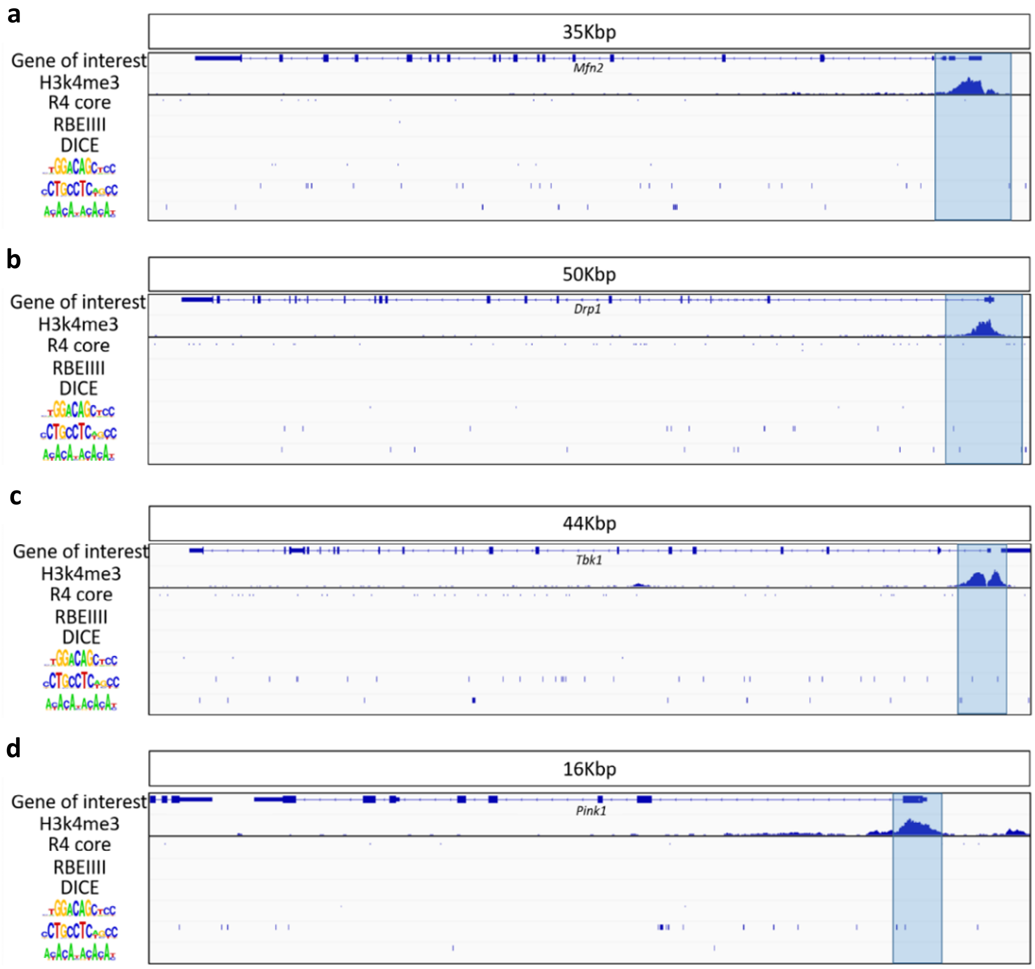

Supplementary Fig. 1

TFII-I canonical and novel binding sequences are adjacent to the regulatory regions of **a** *Mfn2*, **b** *Drp1*, **c** *Tbk1* and **d** *Pink1* in mice, suggesting that TFII-I may directly regulate the expression levels of these genes.

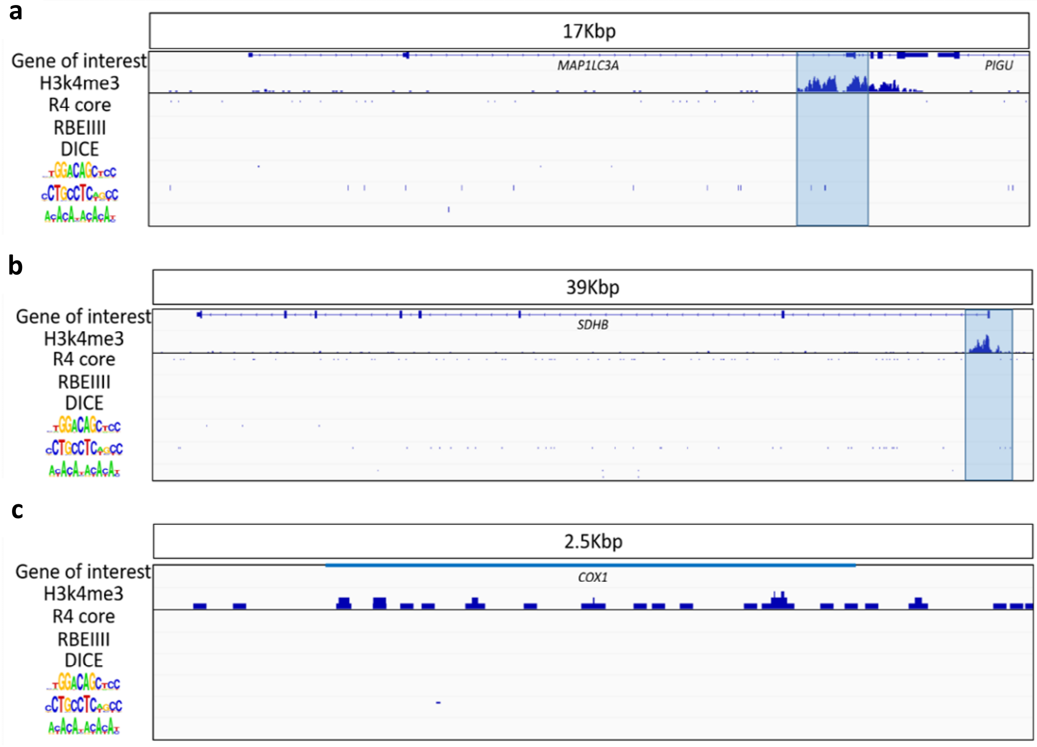

Supplementary Fig. 2

TFII-I canonical and novel binding sequences are adjacent to the regulatory regions of **a** *MAP1LC3A*, **b** *SDHB* and **c** *COX1* in humans, suggesting that TFII-I may directly regulate the expression levels of these genes.

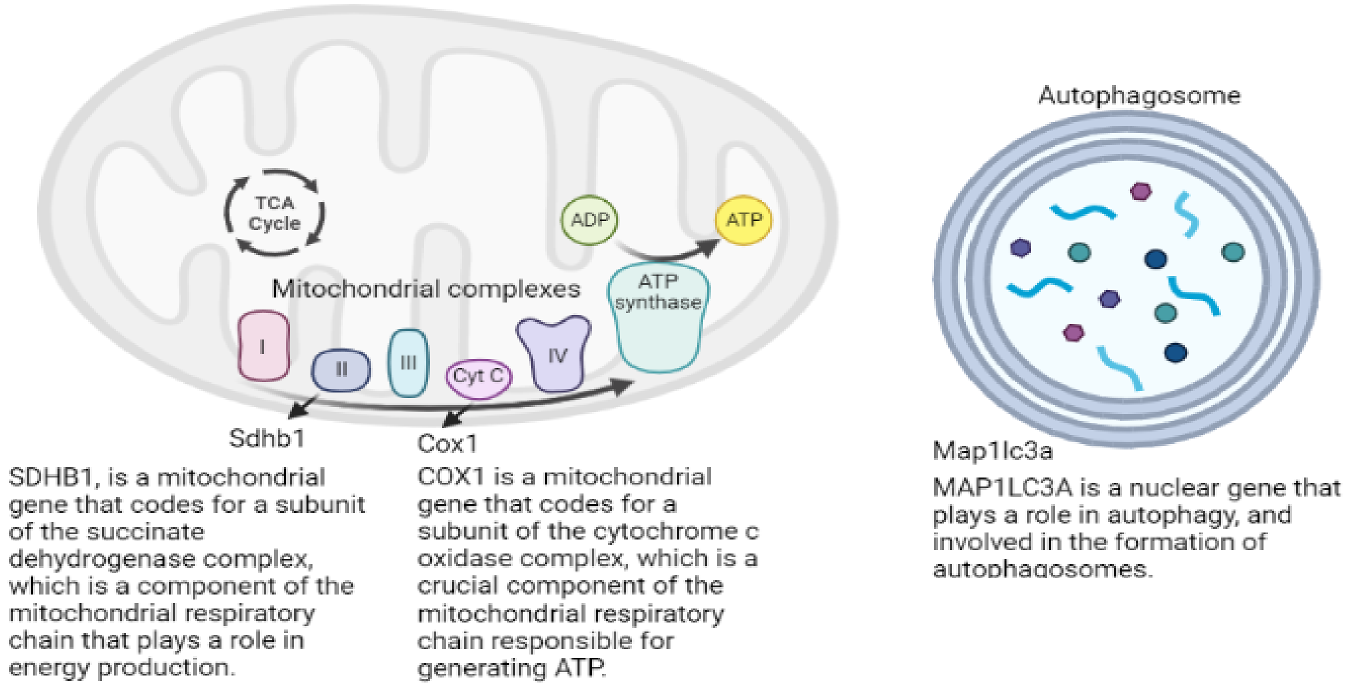

Supplementary Fig. 3

Sdhb1 is an integral component of mitochondrial complex II, playing a crucial role in energy production. Cox1 is a known marker for mitochondrial content and is part of complex V. Map1lc3a, also known as LC3, is associated with autophagy, contributing to the cellular recycling process.

		Mitochondrial.fusion	Pink1.PRKN.mediated.mitophagy	Mitochondrial.biogenesis	Response.to.oxidative.stress	Mitophagy	Mitochondrial.fission	Negative.regulation.of.oxidative.stress.induced.cell.death	Response.to.mitochondrial.depolarisation	Apoptotic.mitochondrial.changes		Structural.constituent.of.cytoskeleton
Barak B. et al., 2019	DW_WS_p<0.01	n.s	n.s	n.s	0.011775	n.s	n.s	0.014858	n.s	0.001837		n.s
	UP_WS_p<0.01	0.01507	0.046614	n.s	n.s	n.s	n.s	0.007546	n.s	0.003588		n.s
	Both_WS_FDR<0.05	0.03267	n.s	n.s	n.s	n.s	n.s	0.03293	n.s	0.004601		n.s
Trangle S. et al., 2023	Hyper methylation	0.030627	n.s	0.049814	n.s	n.s	n.s	0.029205	n.s	n.s		n.s
	Hypo methylation	n.s	n.s	n.s	n.s	n.s	n.s	n.s	n.s	n.s		n.s
Barak B. et al., 2019	Mice.p1	0.009528	0.029649	n.s	n.s	0.049729	0.002227	n.s	0.032302	n.s		n.s
	Mice.1m	0.013456	0.041695	n.s	n.s	n.s	n.s	n.s	0.0454	n.s		n.s

n.s – not significant, Ignored
Color – odds ratio
Number – adj.Pval

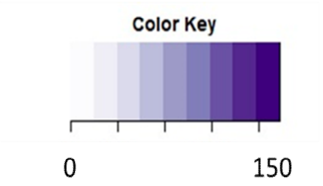

Supplementary Fig. 4

Multi correlation analysis was performed between a set of genes derived from key biological pathways involved in mitochondrial functions (e.g., fusion, fission, autophagy, oxidative stress) and differentially expressed genes (DEGs) or DNA methylation sites from individuals with WS, as compared to controls. Transcriptional data were taken from (i) human brain samples from individuals with WS [1] or (ii) brain samples from mice with *Gtf2i* homozygously deleted from excitatory neurons [1]. DNA methylation data were taken from (iii) human brain samples from individuals with WS [2]. *P* adjusted value (presented in numbers) and odds ratio (color scale) from Fisher's exact were calculated by GeneOverlap package and presented in the heatmap.

Supplementary Fig. 5: uncropped westerns

Molecular weight ladder-

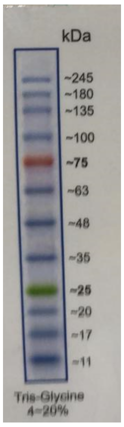

Fig. 5c

Hif1a P1

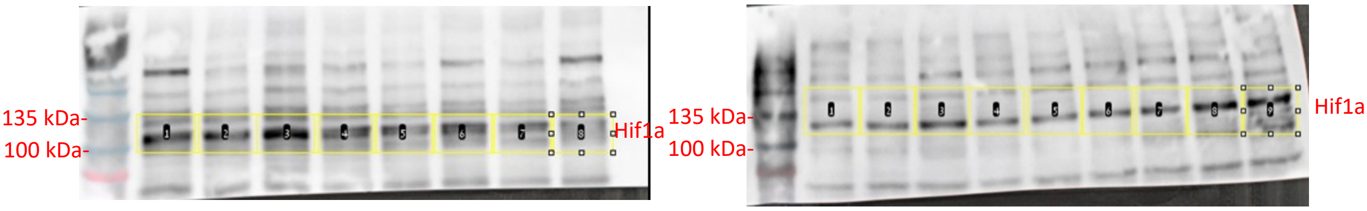

Tubulin

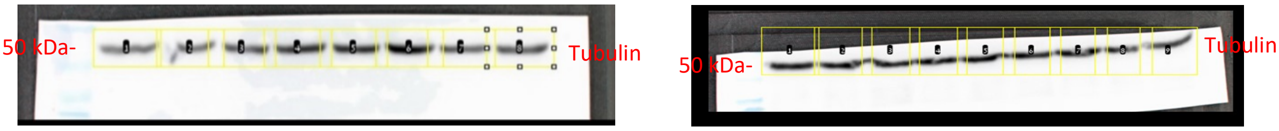

Hif1a P30

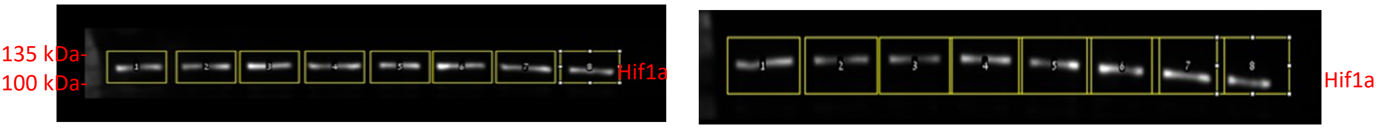

Tubulin

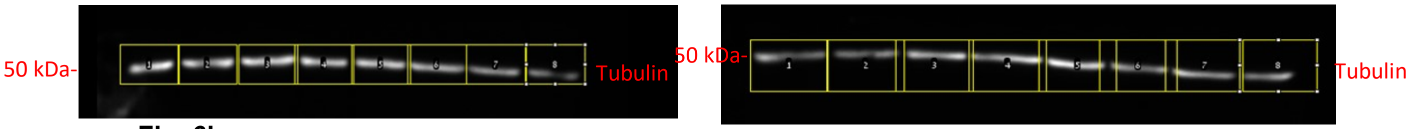

Fig. 6b

LC3 under EBSS starvation in primary neuronal culture

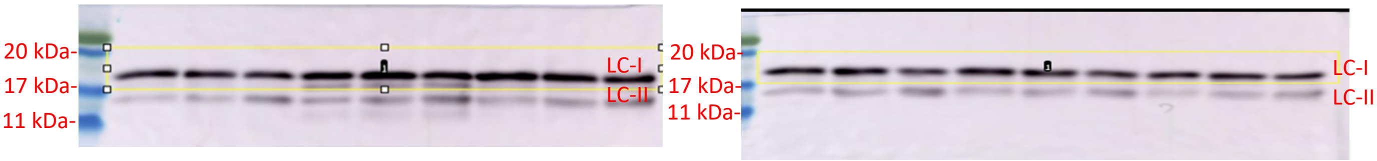

Tubulin

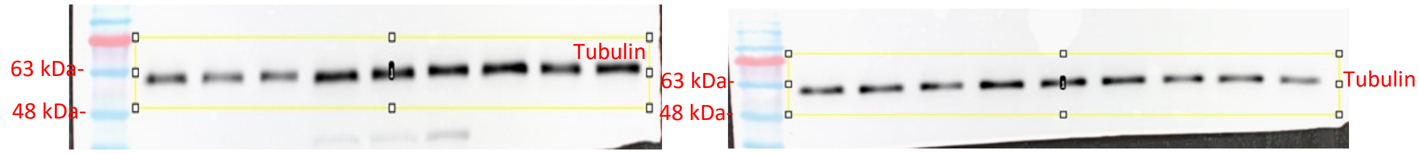

Fig. 6f

Pink1 in primary neuronal culture

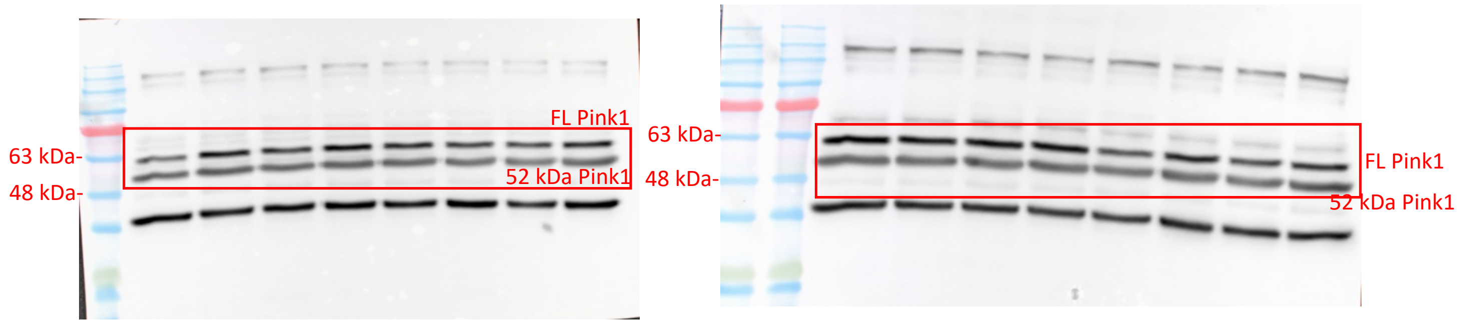

Tubulin

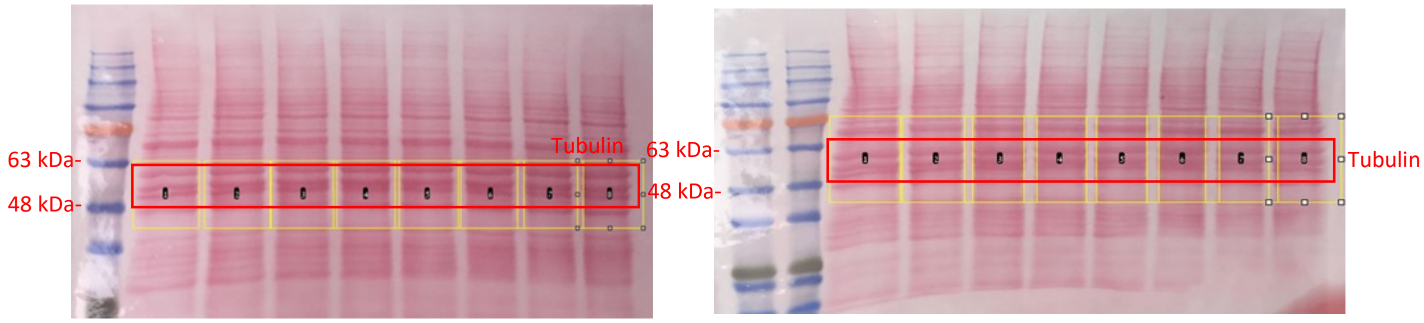

Parkin in primary neuronal culture (part of starvation experiment)

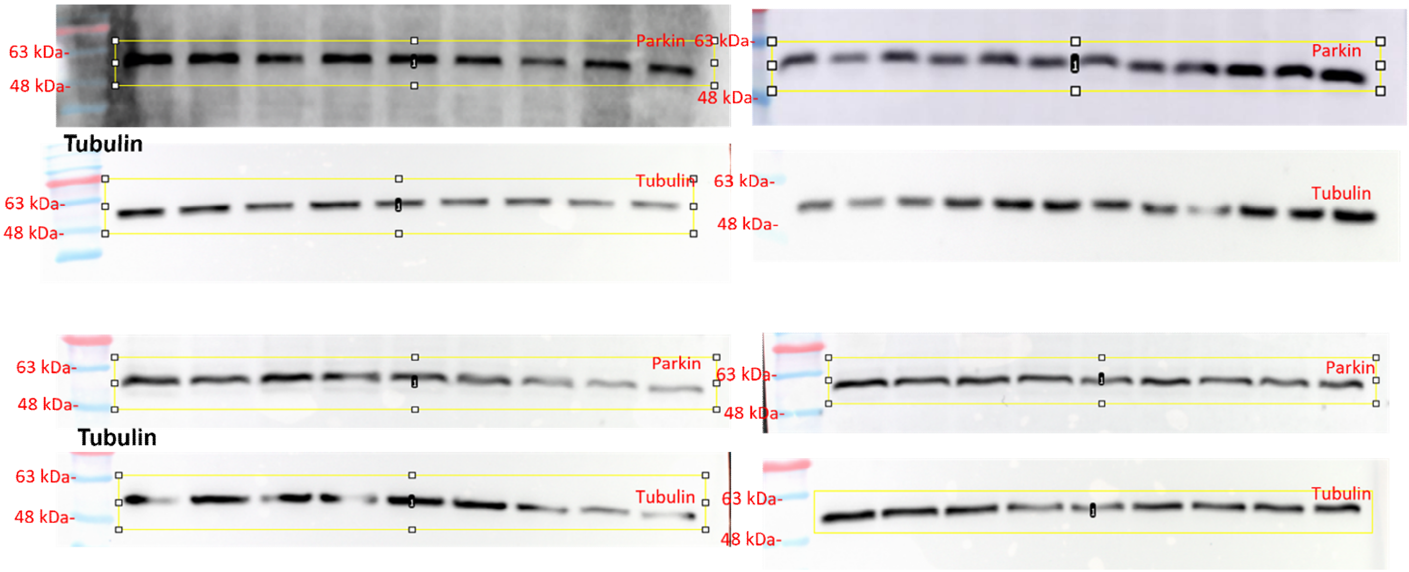

Fig. 7e

Pink1 P1

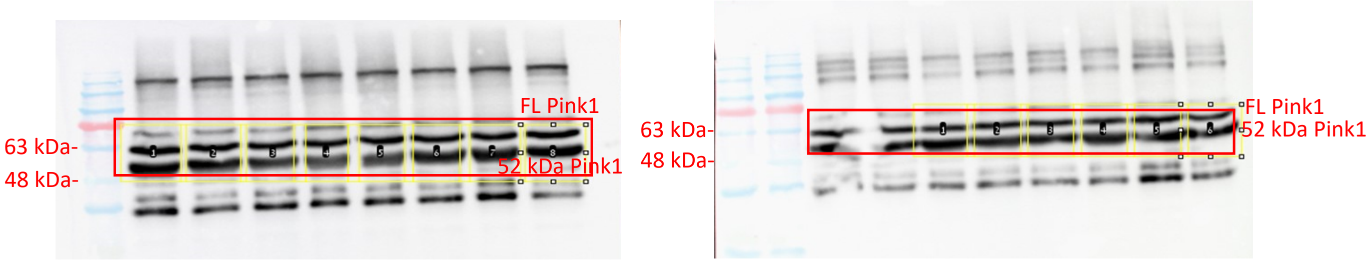

Tubulin

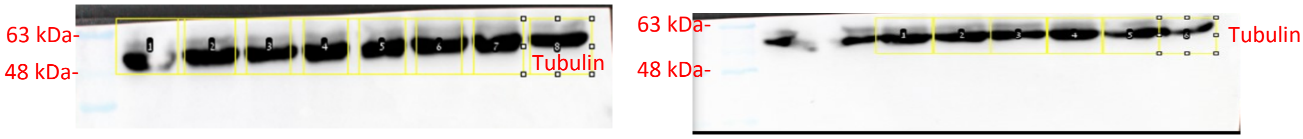

Pink1 P30

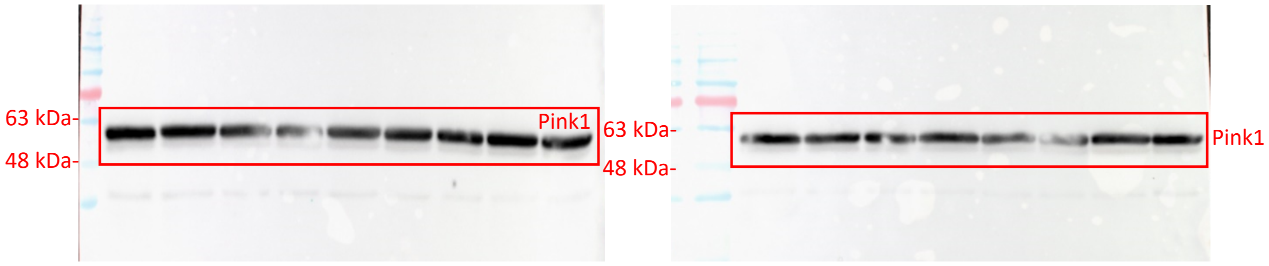

Tubulin

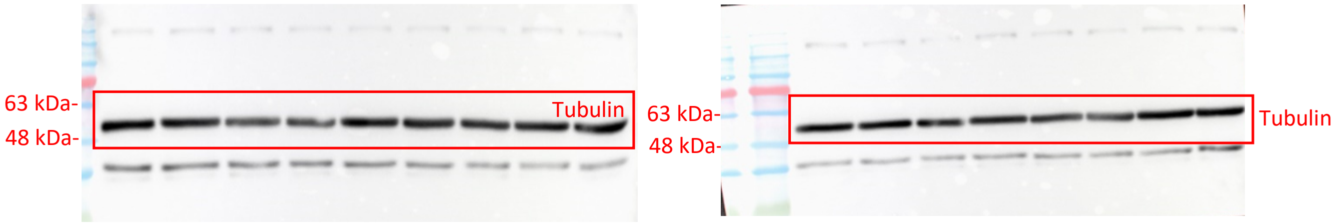

Parkin P1

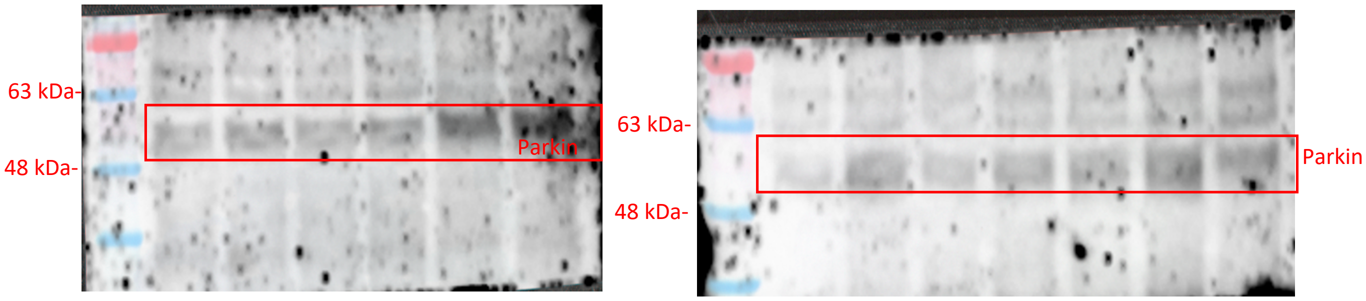

Tubulin

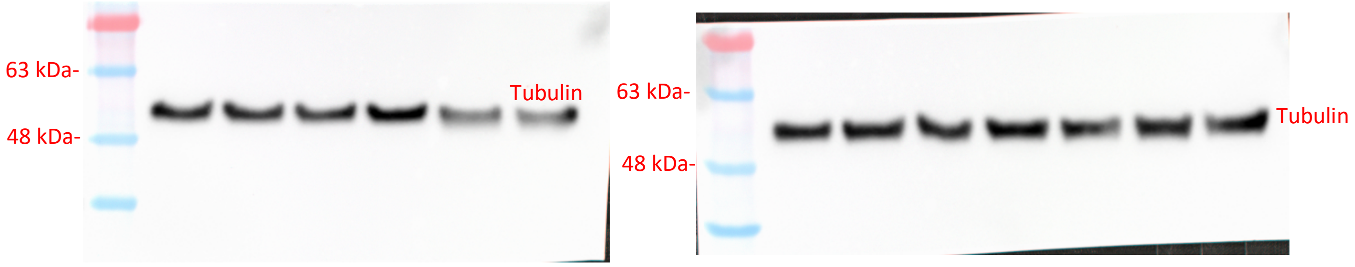

Parkin P30

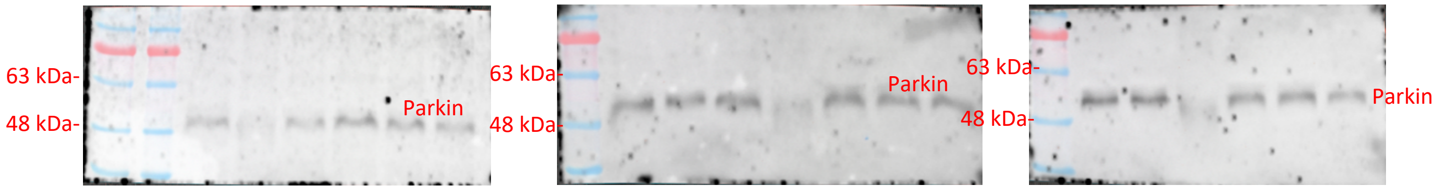

Tubulin

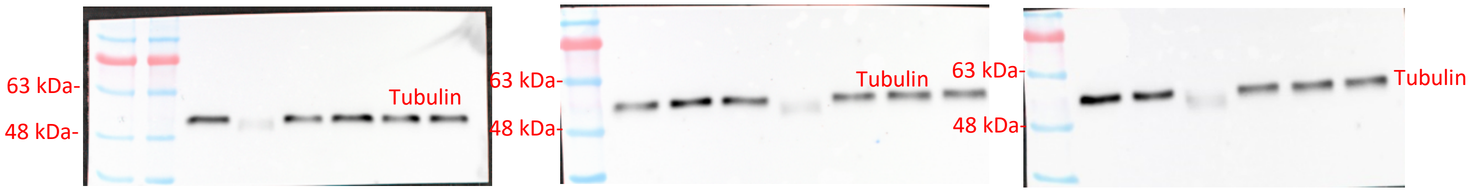

Table 1. SYBR Green RT-PCR primers for mRNA quantification

Origin	Sequence forward	Sequence reverse
Gapdh	GCCTTCCGTGTTCCTACC	CCTCAGTGTAGCCCAAGATG
Drp1	GATTGAAGGAACCGCAAAGTACA	ACAGATTCTAAGGTTCGCCCG
Mfn2	CTGGATGCTGATGTGTTTGTGC	GGAGAGACGCTCACTCACCTTGT
Sdhb	AAGAAGGATGAGTCCCAGGAG	CTTGTCTCCGTTCCACCAGTA
Mtco1	AGAACCCAGTGTCCAGCAAG	CCATCTGTTCCCTCCACATC
Hif1a	GCTCACCATCAGTTATTTACGTGTG	AGCACCATCACAAAGCCATCTAG
Pgc1a	GGACAGTCTCCCCGTGGAT	TCCATCTGTCAGTGCATCAAATG
Jun	TAGCGGAGTCTTAACCCTGCG	CAAAGTTTGTTTAGGAGCGTGCG
LC3	GACCGCTGTAAGGAGGTGC	CTTGACCAACTCGCTCATGTTA
Pink1	CTTATAGGAAAGGGCCCGGATGTCG	GATGATGTTAGGGTGTGGGGCAAGC
Parkin	GCAAACAAGCAACCCTCACCT	GGAGACTGGCACTCACCACTCA
Tbk1	AGACCAGTGATGTGCTTCACCGA	GTTTATAGACCAGTTCATGGAACACAGC

Table 2. Primers used for real-time qPCR (5’→3’)

Origin	Sequence forward	Sequence reverse
MAP1LC3A	CAGCATGGTGAGTGTGTCC	GTCCTCGTCTTTCTCCTGCT
SDHB1	ACATGTGTGGAAGAGGGTAGA	CAACACTCTAGCTTGCACCC
COX1	TCTTACCTCCCTCTCTCCTACTCC	GTTCAACCTGTTCCTGCTCC

HIF1a	GCAAGTCCTCAAAGCACAGT	ATCAGTGGTGGCAGTGGTAG
TUBA1B	CGCCCGTCTCACTGAAGA	GCCAGATGCCAAGTGACAAG

References

1. Barak, B., et al., *Neuronal deletion of Gtf2i, associated with Williams syndrome, causes behavioral and myelin alterations rescuable by a remyelinating drug.* Nature Neuroscience, 2019. **22**(5): p. 700-708.
2. Trangle, S.S., et al., *In individuals with Williams syndrome, dysregulation of methylation in non-coding regions of neuronal and oligodendrocyte DNA is associated with pathology and cortical development.* Molecular Psychiatry, 2023. **28**(3): p. 1112-1127.
