# Supplementary material for: Neuronal Gtf2i deletion alters mitochondrial and autophagic properties
Source: Commun Biol. 2023 Dec 14;6:1269. doi: 10.1038/s42003-023-05612-5 (PMC10721858; doi:10.1038/s42003-023-05612-5)
Supplement: Supplementary file 4 — Reporting Summary [file 42003_2023_5612_MOESM4_ESM.pdf]

nature portfolio

Corresponding author(s): Boaz Barak

Last updated by author(s): Nov 1, 2023

Reporting Summary

Nature Portfolio wishes to improve the reproducibility of the work that we publish. This form provides structure for consistency and transparency in reporting. For further information on Nature Portfolio policies, see our Editorial Policies and the Editorial Policy Checklist.

Statistics

For all statistical analyses, confirm that the following items are present in the figure legend, table legend, main text, or Methods section.

n/a	Confirmed	
☐	☒	The exact sample size (*n*) for each experimental group/condition, given as a discrete number and unit of measurement
☐	☒	A statement on whether measurements were taken from distinct samples or whether the same sample was measured repeatedly
☐	☒	The statistical test(s) used AND whether they are one- or two-sided<br>*Only common tests should be described solely by name; describe more complex techniques in the Methods section.*
☐	☒	A description of all covariates tested
☐	☒	A description of any assumptions or corrections, such as tests of normality and adjustment for multiple comparisons
☐	☒	A full description of the statistical parameters including central tendency (e.g. means) or other basic estimates (e.g. regression coefficient) AND variation (e.g. standard deviation) or associated estimates of uncertainty (e.g. confidence intervals)
☐	☒	For null hypothesis testing, the test statistic (e.g. *F*, *t*, *r*) with confidence intervals, effect sizes, degrees of freedom and *P* value noted<br>*Give P values as exact values whenever suitable.*
☒	☐	For Bayesian analysis, information on the choice of priors and Markov chain Monte Carlo settings
☒	☐	For hierarchical and complex designs, identification of the appropriate level for tests and full reporting of outcomes
☒	☐	Estimates of effect sizes (e.g. Cohen's *d*, Pearson's *r*), indicating how they were calculated

Our web collection on statistics for biologists contains articles on many of the points above.

Software and code

Policy information about availability of computer code

Data collection	FIJI
Data analysis	FIJI, Prism

For manuscripts utilizing custom algorithms or software that are central to the research but not yet described in published literature, software must be made available to editors and reviewers. We strongly encourage code deposition in a community repository (e.g. GitHub). See the Nature Portfolio guidelines for submitting code & software for further information.

Data

Policy information about availability of data

All manuscripts must include a data availability statement. This statement should provide the following information, where applicable:

- Accession codes, unique identifiers, or web links for publicly available datasets
- A description of any restrictions on data availability
- For clinical datasets or third party data, please ensure that the statement adheres to our policy

All data are included in the manuscript and the supplementary information and will be accessible to anyone interested upon reasonable request. All uncropped blots images generated and analyzed during the current study are available in the supplementary information. All materials are commercialized and will be available upon reasonable request.

Research involving human participants, their data, or biological material

Policy information about studies with human participants or human data. See also policy information about sex, gender (identity/presentation), and sexual orientation and race, ethnicity and racism.

Reporting on sex and gender	This study included 9 human brain samples from frontal cortex BA9 sections from 5 males and 4 females. Sex and gender were not considered in the study design.
Reporting on race, ethnicity, or other socially relevant groupings	The study races included 9 white, 2 black or African-American, and 1 Indian Subcontinent.
Population characteristics	The ages in this study were- 17, 24, 34, 34, 42, 42, 42, 45, 45.<br>6 samples were donated by control healthy brains, and 3 from Williams syndrome patients.
Recruitment	*Describe how participants were recruited. Outline any potential self-selection bias or other biases that may be present and how these are likely to impact results.*
Ethics oversight	*Identify the organization(s) that approved the study protocol.*

Note that full information on the approval of the study protocol must also be provided in the manuscript.

Field-specific reporting

Please select the one below that is the best fit for your research. If you are not sure, read the appropriate sections before making your selection.

☒ Life sciences ☐ Behavioural & social sciences ☐ Ecological, evolutionary & environmental sciences

For a reference copy of the document with all sections, see nature.com/documents/nr-reporting-summary-flat.pdf

Life sciences study design

All studies must disclose on these points even when the disclosure is negative.

Sample size	No statistical methods were used to predetermine sample size. Sample size used is based on common and accepted size in the field.
Data exclusions	Outliers were determined via the extreme studentized deviate (ESD) method.
Replication	Results were replicated by using more than one batch of test animals or were validated by other technical means and methods
Randomization	Experiments were randomized: samples order, genotypes used and samples loading were all randomized and counterbalanced
Blinding	All the investigators were blinded to genotype during experiments and outcome assessment

Reporting for specific materials, systems and methods

We require information from authors about some types of materials, experimental systems and methods used in many studies. Here, indicate whether each material, system or method listed is relevant to your study. If you are not sure if a list item applies to your research, read the appropriate section before selecting a response.

Materials & experimental systems

n/a	Involved in the study	
☐	☒	Antibodies
☒	☐	Eukaryotic cell lines
☒	☐	Palaeontology and archaeology
☐	☒	Animals and other organisms
☒	☐	Clinical data
☒	☐	Dual use research of concern
☒	☐	Plants

Methods

n/a	Involved in the study	
☒	☐	ChIP-seq
☐	☒	Flow cytometry
☒	☐	MRI-based neuroimaging

Antibodies

Antibodies used	Commercial antibodies used included anti-TFII-I (1:1000; CST-4562S, Cell signaling), anti-CaM Kinase IIa (1:400; 50049, Cell signaling), anti-Iba1 (1:500; 234 006, SYSY) anti-S100B (1:600; S-2532, Sigma-Aldrich) and anti-Gad67 (1:1000; MAB5406, Millipore) antibodies.
Validation	All antibodies were tested for specific and accurate function, and were used in other articles successfully

Animals and other research organisms

Policy information about studies involving animals; ARRIVE guidelines recommended for reporting animal research, and Sex and Gender in Research

Laboratory animals	All mice were on C57Bl/6 background, all males, and ages are described in materials and methods section
Wild animals	N/A
Reporting on sex	All test mice were males. Therefore, the limitations of this article arise from the absence of considering the sex of the mice in the experimental design, potentially overlooking gender-specific differences in the results.
Field-collected samples	N/A
Ethics oversight	All experimental protocols conformed to the guidelines of the Institutional Animal Care and Use Committee of Tel Aviv University, Tel Aviv, Israel.

Note that full information on the approval of the study protocol must also be provided in the manuscript.

Plants

Seed stocks	N/A
Novel plant genotypes	N/A
Authentication	N/A

Flow Cytometry

Plots

Confirm that:

- [x] The axis labels state the marker and fluorochrome used (e.g. CD4-FITC).
- [x] The axis scales are clearly visible. Include numbers along axes only for bottom left plot of group (a 'group' is an analysis of identical markers).
- [x] All plots are contour plots with outliers or pseudocolor plots.
- [x] A numerical value for number of cells or percentage (with statistics) is provided.

Methodology

Sample preparation	For flow cytometry analysis of DCF staining, 250,000 cells from primary neuronal cultures were plated on matrigel-coated 6-well plates. After 14 days, the cells were washed and loaded with 10uM of the dye (Sigma-Aldrich, D6883) for 30 min at 37°C. After washing to remove excess dye, the cells were suspended in trypsin for 5 min at 37°C and then in PBS and centrifuged. As a control, 1 µM of H_2O_2 was introduced to the cells for 2 h at 37°C.
Instrument	Stratedigm S1000EXi flow cytometry
Software	The data was analyzed via the Cyflogic software
Cell population abundance	N/A

Gating strategy | N/A

☐ Tick this box to confirm that a figure exemplifying the gating strategy is provided in the Supplementary Information.
